# Supplementary material for: Dietary Zinc Supplemented in Organic Form Affects the Expression of Inflammatory Molecules in Swine Intestine
Source: Animals (Basel). 2023 Aug 4;13(15):2519. doi: 10.3390/ani13152519 (PMC10417787; doi:10.3390/ani13152519)
Supplement: Supplementary file 1 [file animals-13-02519-s001.zip › animals-2465240-supplementary.pdf]

Supplementary Material

Supplementary Table S1. Weight gain of pigs in different treatment groups

Item	Diet					SEM	*p*-value
	ZnR group	Zn50	Zn100	LQ50	LQ100		
**ADG**, kg/d	1.20	1.19	1.21	1.16	1.12	0.03	0.20
**ADFI**, kg/d	2.96	2.82	2.81	2.78	2.66		

ADG: Average daily gain; ADFI: Average daily feed intake
The results are least square means of nine pigs treated with the same diet. ZnR: ZnR: (Zn-free vitamin-mineral mix + 0 ppm Zn), Zn50: (ZnR with 50 ppm Zn from $ZnCl_2$), Zn100: (ZnR with 100 ppm Zn from $ZnCl_2$), LQ50: (ZnR with 50 ppm Zn from LQ-Zn), LQ100: (ZnR with 100 ppm Zn from LQ-Zn). SEM means standard error.

Supplementary Table S2. Differentially expressed genes in Zn50 group vs LQ50 group

Transcript ID	Gene description	Log2 fold change	*p*-value
ENSSSCT00000018609.3	TNFAIP3 interacting protein 1	-3.513225166	0.006398767
ENSSSCT00000064304.1	tetratricopeptide repeat domain 38	-3.407471637	0.005685538
ENSSSCT00000024467.2	lithostathine-like	-3.319263217	5.73E-05
ENSSSCT00000010090.3	ATP-binding cassette sub-family G member 2-like	-3.287787957	0.00120746
ENSSSCT00000044646.1	TATA-box binding protein associated factor, RNA polymerase I subunit A	-3.070691954	0.001891867
ENSSSCT00000009101.3	exocyst complex component 6B	-3.047415705	0.037993933
ENSSSCT00000055096.1	XK related 9	-2.858200886	0.024425041
ENSSSCT00000062907.1	N/A	-2.755164061	0.028145634
ENSSSCT00000016255.3	RAB30, member RAS oncogene family	-2.709060135	0.004028253
ENSSSCT00000046789.1	interferon alpha and beta receptor subunit 2	-2.702254735	0.003796125
ENSSSCT00000015136.3	epidermal growth factor receptor pathway substrate 15 like 1	-2.701595575	0.004147611
ENSSSCT00000038998.1	myoferlin	-2.701355041	0.039874082
ENSSSCT00000006983.3	intelectin-2-like	-2.670138395	0.025536689
ENSSSCT00000033063.2	integrator complex subunit 6 like	-2.641544482	0.013753713
ENSSSCT00000061466.1	transcription elongation factor A N-terminal and central domain containing	-2.582828032	0.011264154
ENSSSCT00000044468.1	tetratricopeptide repeat domain 39A	-2.559267689	0.005928743
ENSSSCT00000035464.2	guanylate-binding protein 6	-2.558786335	0.002839816
ENSSSCT00000063298.1	N(alpha)-acetyltransferase 16, NatA auxiliary subunit	-2.529171199	0.023560128
ENSSSCT00000054433.1	toll like receptor 2	-2.504011119	0.02069795
ENSSSCT00000023711.2	UBX domain protein 11	-2.468216068	0.032735322
ENSSSCT00000057593.1	proteasomal ATPase associated factor 1	-2.456549021	0.015438489
ENSSSCT00000031489.2	phospholipid scramblase 1	-2.446918771	0.000877547
ENSSSCT00000040813.1	eukaryotic translation initiation factor 5	-2.439114324	0.020221238
ENSSSCT00000049093.1	DDB1 and CUL4 associated factor 5	-2.286328006	0.043020524
ENSSSCT00000057064.1	polyhomeotic homolog 3	-2.229829076	0.027724405
ENSSSCT00000017706.3	SP110 nuclear body protein	-2.227555279	0.042510888
ENSSSCT00000037785.1	sortilin related VPS10 domain containing receptor 2	-2.141693677	0.021000471
ENSSSCT00000060349.1	chromosome 1 C18orf25 homolog	-2.101480495	0.039344637
ENSSSCT00000060480.1	N-glycanase 1	-2.097030826	0.02951043

ENSSSCT00000039871.1	N/A	-2.09420064	0.033181564
ENSSSCT00000035298.2	neurocalcin delta	-2.059552954	0.021374781
ENSSSCT00000002211.3	syntaxin binding protein 6	-2.027004385	0.00099521
ENSSSCT00000063642.1	ankyrin repeat and FYVE domain containing 1	-1.976716253	0.048119165
ENSSSCT00000034011.2	casein kinase 2 beta	-1.961755156	0.046795073
ENSSSCT00000009314.3	N/A	-1.956201038	0.047200924
ENSSSCT00000054913.1	solute carrier family 12 member 6	-1.95338103	0.00405913
ENSSSCT00000047282.1	dystrobrevin binding protein 1	-1.945955034	0.037363869
ENSSSCT00000010484.3	transmembrane and coiled-coil domains 3	-1.936765683	0.020843787
ENSSSCT00000026637.2	coiled-coil domain containing 66	-1.921742982	0.041569272
ENSSSCT00000026212.2	lithostathine-like	-1.818799507	0.016205807
ENSSSCT00000054142.1	autophagy related 7	-1.804566945	0.024344093
ENSSSCT00000066043.1	serine/threonine kinase 40	-1.804525302	0.038768021
ENSSSCT00000059075.1	N/A	-1.802459613	0.020857056
ENSSSCT00000034952.2	lithostathine-like	-1.791859808	0.010909468
ENSSSCT00000016435.3	apolipoprotein C3	-1.791574256	0.012770675
ENSSSCT00000065983.1	mitochondrial ribosomal protein L11	-1.763632457	0.038111723
ENSSSCT00000044987.1	ubiquitin specific peptidase 34	-1.754065789	0.002345463
ENSSSCT00000032794.2	guanylate binding protein 1, interferon-inducible	-1.686142933	0.023665061
ENSSSCT00000061852.1	apolipoprotein A4	-1.659064515	0.014428336
ENSSSCT00000035960.2	deleted in malignant brain tumors 1	-1.655904363	0.00066116
ENSSSCT00000016434.3	apolipoprotein A4	-1.635163315	0.011545978
ENSSSCT00000032333.2	N/A	-1.603767545	0.039138766
ENSSSCT00000008980.3	lectin, mannose binding 2 like	-1.583399507	0.028262828
ENSSSCT00000029033.2	synaptotagmin 12	-1.575029585	0.022389225
ENSSSCT00000066145.1	TANK binding kinase 1	-1.534617483	0.04989232
ENSSSCT00000054422.1	apolipoprotein C3	-1.531618049	0.037561727
ENSSSCT00000063395.1	solute carrier family 30 member 5	-1.517675668	0.012757594
ENSSSCT00000063000.1	STT3A, catalytic subunit of the oligosaccharyltransferase complex	-1.507933083	0.038356762
ENSSSCT00000057863.1	3'(2'), 5'-bisphosphate nucleotidase 1	-1.49302696	0.006839811
ENSSSCT00000035208.2	FYN binding protein 1	-1.480273781	0.022411796
ENSSSCT00000006736.3	carbonic anhydrase 13	-1.469686042	0.022482409
ENSSSCT00000051608.1	N/A	-1.464791334	0.019734326

ENSSSCT00000052291.1	centromere protein O	-1.456325541	0.047810632
ENSSSCT00000011816.2	beta-1,3-galactosyltransferase 2	-1.407720665	0.029509267
ENSSSCT00000036737.1	N/A	-1.393990749	0.049320853
ENSSSCT00000065002.1	tryptophanyl-tRNA synthetase	-1.392329044	3.28E-06
ENSSSCT00000037379.1	RCC1 and BTB domain containing protein 1	-1.372247145	0.021357576
ENSSSCT00000009132.3	glutamine--fructose-6-phosphate transaminase 1	-1.362890813	0.045600934
ENSSSCT00000049047.1	N/A	-1.336488793	0.028832637
ENSSSCT00000053084.1	calpastatin	-1.324185214	0.002905149
ENSSSCT00000053953.1	KIAA1211 ortholog	-1.3227409	0.029511612
ENSSSCT00000016117.2	N/A	-1.301216372	0.020812321
ENSSSCT00000065180.1	nitric oxide synthase 2	-1.291739279	4.92E-05
ENSSSCT00000051826.1	U2 snRNP associated SURP domain containing	-1.289737391	0.045570696
ENSSSCT00000058188.1	SLC9A3 regulator 1	-1.28684978	0.045798532
ENSSSCT00000048959.1	transmembrane channel like 5	-1.280668176	0.046981769
ENSSSCT00000013421.4	ubiquitin specific peptidase 11	-1.25580422	0.000870226
ENSSSCT00000050257.1	GTPase, very large interferon inducible 1	-1.248787103	0.031632498
ENSSSCT00000043753.1	SPT2 chromatin protein domain containing 1	-1.236569435	0.007143788
ENSSSCT00000004701.2	golgi associated PDZ and coiled-coil motif containing	-1.223227265	0.021384478
ENSSSCT00000062032.1	semaphorin 4B	-1.209040391	0.039468871
ENSSSCT00000064480.1	transcription elongation regulator 1	-1.199824983	0.045938196
ENSSSCT00000058969.1	guanosine monophosphate reductase	-1.190638852	0.047710383
ENSSSCT00000037704.1	N/A	-1.168553776	0.00682783
ENSSSCT00000014795.3	crumbs 3, cell polarity complex component	-1.162856107	0.031142832
ENSSSCT00000045468.1	globoside alpha-1,3-N-acetylgalactosaminyltransferase 1 (FORS blood group)	-1.146225053	0.006839842
ENSSSCT00000059647.1	UBA domain containing 2	-1.142174216	0.026770579
ENSSSCT00000037353.1	N/A	-1.139767175	0.000692293
ENSSSCT00000002141.3	promyelocytic leukemia	-1.106962744	0.005489652
ENSSSCT00000056748.1	galectin 9	-1.083223382	0.001638694
ENSSSCT00000065175.1		-1.082455311	0.013540924
ENSSSCT00000006270.3	argininosuccinate synthase 1	-1.078354252	9.58E-05
ENSSSCT00000038306.1	N/A	-1.075517794	0.041028573
ENSSSCT00000056308.1	Rho GTPase activating protein 8	-1.069906143	0.048570371

ENSSSCT00000002555.4	arginase 2	-1.009382594	0.010812847
ENSSSCT00000031638.2	lysine demethylase 4A	-0.983045105	0.011408154
ENSSSCT00000044710.1	BCL2 like 15	-0.971493517	0.010757749
ENSSSCT00000003489.2	fucosyltransferase 2	-0.934767883	0.040988873
ENSSSCT00000053279.1	solute carrier family 6 member 9	-0.934472408	0.031586038
ENSSSCT00000030526.2	guanylate binding protein 2, interferon-inducible	-0.931192225	0.034238209
ENSSSCT00000039827.1	MYC associated factor X	-0.903238528	0.022952307
ENSSSCT00000006932.3	aldehyde dehydrogenase 9 family member A1	-0.89042723	0.005970285
ENSSSCT00000012365.3	SNF related kinase	-0.859662076	0.047993214
ENSSSCT00000026608.2	DNA primase subunit 1	-0.85618351	0.036719484
ENSSSCT00000001595.4	butyrophilin-like protein 1	-0.851065451	0.017259806
ENSSSCT00000041931.1	N/A	-0.815306001	0.023573408
ENSSSCT00000015261.3	LY6/PLAUR domain containing 8	-0.814254963	5.80E-09
ENSSSCT00000044789.1	3-oxoacid CoA-transferase 1	-0.807310535	0.01832443
ENSSSCT00000059131.1	BCL2 like 15	-0.793811466	0.016477411
ENSSSCT00000061681.1	argininosuccinate synthase 1	-0.792777389	0.007070418
ENSSSCT00000057681.1	sideroflexin 1	-0.790469707	0.026527453
ENSSSCT00000035232.2	interferon regulatory factor 7	-0.766214797	0.022302993
ENSSSCT00000059937.1	tetraspanin 1	-0.762820621	0.029630716
ENSSSCT00000019325.3	nitric oxide synthase 2	-0.753143435	0.004341459
ENSSSCT00000014648.3	adrenomedullin	-0.743109483	0.039455722
ENSSSCT00000018765.3	CD300c molecule	-0.735835827	0.046592162
ENSSSCT00000046926.1	legumain	-0.698455494	0.030446925
ENSSSCT00000028621.2	interleukin 18 binding protein	-0.660321096	0.034057051
ENSSSCT00000025376.2	basic leucine zipper ATF-like transcription factor 2	-0.659117381	0.00373003
ENSSSCT00000011756.3	DEAH-box helicase 32 (putative)	-0.642316456	0.040386419
ENSSSCT00000010934.3	adaptor related protein complex 1 subunit beta 1	-0.628839435	0.035521366
ENSSSCT00000005528.3	ADP ribosylation factor 6	-0.605611648	0.018145748
ENSSSCT00000016571.3	hepatic and glial cell adhesion molecule	-0.604874192	0.020908795
ENSSSCT00000002537.3	glutathione peroxidase 2	-0.596970565	0.005450085
ENSSSCT00000061945.1	guanylate binding protein 1, interferon-inducible	-0.593047916	0.038170414
ENSSSCT00000024524.2	protein phosphatase 4 regulatory subunit 1	-0.589791068	0.00759609

ENSSSCT00000063487.1	heat shock protein family A (Hsp70) member 8	-0.589643274	0.007110243
ENSSSCT00000063260.1	poly(ADP-ribose) polymerase family member 9	-0.585389931	0.019636658
ENSSSCT00000037199.1	dual specificity phosphatase 6	-0.582386413	0.028996624
ENSSSCT00000065553.1	uridine phosphorylase 1	-0.575523213	0.016407208
ENSSSCT00000045547.1	actin gamma 1	-0.573243875	0.01437517
ENSSSCT00000052265.1	erythrocyte membrane protein band 4.1 like 3	-0.573181494	0.023918264
ENSSSCT00000027977.2	trefoil factor 2	-0.572521071	0.027259813
ENSSSCT00000029497.2	7-dehydrocholesterol reductase	-0.562336747	0.016346651
ENSSSCT00000057292.1	carbohydrate sulfotransferase 4	-0.561592532	0.024717759
ENSSSCT00000055407.1	interferon-induced very large GTPase 1-like	-0.558752835	0.028201173
ENSSSCT00000014465.3	ADP ribosylation factor GTPase activating protein 2	-0.533283377	0.021315584
ENSSSCT00000044432.1	serine/threonine kinase 24	-0.523249697	0.035273875
ENSSSCT00000058772.1	interferon induced transmembrane protein 3	-0.517224236	0.040538953
ENSSSCT00000003977.3	sphingomyelin phosphodiesterase acid like 3B	-0.508870651	0.041962666
ENSSSCT00000062753.1	clathrin light chain A	-0.490938187	0.020418147
ENSSSCT00000004323.3	tetraspanin 1	-0.489896209	0.020192235
ENSSSCT00000064890.1	NCK adaptor protein 2	-0.485029284	0.036809331
ENSSSCT00000064525.1	actin related protein 2/3 complex subunit 2	-0.478788575	0.04589156
ENSSSCT00000024216.2	solute carrier family 43 member 2	-0.477561962	0.029734379
ENSSSCT00000048970.1	glucose-6-phosphate dehydrogenase	-0.473520413	0.006427094
ENSSSCT00000035478.2	tripartite motif containing 25	-0.472176948	0.033794329
ENSSSCT00000053991.1	RAB7A, member RAS oncogene family	-0.471696306	0.006151996
ENSSSCT00000051775.1	RALY heterogeneous nuclear ribonucleoprotein	-0.466360579	0.042611493
ENSSSCT00000040939.1	oxoglutarate dehydrogenase	-0.459414652	0.040511157
ENSSSCT00000053446.1	mitogen-activated protein kinase 3	-0.436784957	0.019884309
ENSSSCT00000001103.3	serpin family B member 1	-0.431825186	0.023013316
ENSSSCT00000005376.2	carnosine dipeptidase 2	-0.427471662	0.040027062
ENSSSCT00000053600.1	Rho GTPase activating protein 17	-0.42186694	0.042575422
ENSSSCT00000007584.4	guanylate binding protein 1, interferon-inducible	-0.419300588	0.033518969
ENSSSCT00000055437.1	protein phosphatase 2 phosphatase activator	-0.41019122	0.017548076
ENSSSCT00000027356.2	leucine aminopeptidase 3	-0.409948977	0.021247107

ENSSSCT00000028713.2	UDP-galactose-4-epimerase	-0.40878454	0.013875496
ENSSSCT00000063535.1	citrate synthase	-0.406726351	0.028983042
ENSSSCT00000055817.1	enolase 1	-0.399048497	0.028455401
ENSSSCT00000000402.3	sulfite oxidase	-0.381169379	0.029924349
ENSSSCT00000007975.3	transient receptor potential cation channel subfamily C member 4 associated protein	-0.381019925	0.048625695
ENSSSCT00000011231.3	pyrophosphatase (inorganic) 1	-0.375733023	0.026911904
ENSSSCT00000003210.3	RNA binding motif protein 42	-0.373672255	0.024348326
ENSSSCT00000022886.2	phosphatidylinositol transfer protein alpha	-0.363712051	0.003729557
ENSSSCT00000057321.1	hydroxyacyl-CoA dehydrogenase	-0.361425929	0.026782034
ENSSSCT00000024290.2	sphingosine kinase 2	-0.358070605	0.048560362
ENSSSCT00000014210.3	calpain 1	-0.357990527	0.041495478
ENSSSCT00000039383.1	LIM and SH3 protein 1	-0.35473195	0.032752442
ENSSSCT00000041757.1	proteasome 26S subunit, non-ATPase 4	-0.349263408	0.028358824
ENSSSCT00000007592.3	SH3 domain containing GRB2 like, endophilin B1	-0.347255437	0.002350515
ENSSSCT00000008551.3	Tu translation elongation factor, mitochondrial	-0.345766595	0.020857629
ENSSSCT00000044242.1	adhesion regulating molecule 1	-0.317814393	0.016438895
ENSSSCT00000031590.2	calpain small subunit 1	-0.315964885	0.01721292
ENSSSCT00000042867.1	Rho GDP dissociation inhibitor alpha	-0.314876691	0.041768745
ENSSSCT00000057478.1	signal transducer and activator of transcription 3	-0.312505859	0.025714627
ENSSSCT00000065139.1	actin beta	-0.309436761	0.032958247
ENSSSCT00000003774.3	phosphogluconate dehydrogenase	-0.278721325	0.016229913
ENSSSCT00000049038.1	chromosome 7 C6orf106 homolog	-0.278380998	0.033840007
ENSSSCT00000018738.3	Jupiter microtubule associated homolog 1	-0.276309267	0.01631853
ENSSSCT00000030514.2	protein phosphatase 2 scaffold subunit Aalpha	-0.27527631	0.045309463
ENSSSCT00000002075.3	isocitrate dehydrogenase (NADP(+)) 2, mitochondrial	-0.267622997	0.016973979
ENSSSCT00000019298.3	proteasome 26S subunit, non-ATPase 11	-0.262026014	0.046112554
ENSSSCT00000013443.4	proteolipid protein 2	-0.247340567	0.030318143
ENSSSCT00000053199.1	JunD proto-oncogene, AP-1 transcription factor subunit	-0.228895718	0.048169312
ENSSSCT00000039540.1	Rac family small GTPase 1	-0.215540073	0.033899122
ENSSSCT00000054290.1	synaptophysin like 1	0.301988176	0.006451118
ENSSSCT00000059250.1	sorting nexin 2	0.338186961	0.045986845

ENSSSCT00000017523.3	CDC like kinase 1	0.350563648	0.014894947
ENSSSCT00000049560.1	anaphase promoting complex subunit 13	0.417062419	0.031848228
ENSSSCT00000005728.3	distal membrane arm assembly complex 1	0.419203473	0.005244475
ENSSSCT00000025147.2	histone cluster 1, H2bd	0.474219508	0.032318089
ENSSSCT00000060390.1	translocase of inner mitochondrial membrane 10B	0.525571131	0.025590746
ENSSSCT00000036085.2	interleukin 18	0.527802131	0.025522583
ENSSSCT00000007500.3	solute carrier family 25 member 24	0.591028289	0.017308112
ENSSSCT00000019677.1	cytochrome c oxidase subunit III	0.68364574	0.041998449
ENSSSCT00000019673.1	cytochrome c oxidase subunit II	0.685031467	0.037485583
ENSSSCT00000019679.3	NADH dehydrogenase subunit 3	0.696227699	0.03615784
ENSSSCT00000019675.1	ATP synthase F0 subunit 8	0.72406022	0.043966946
ENSSSCT00000019670.1	cytochrome c oxidase subunit I	0.731576392	0.042510767
ENSSSCT00000006801.3	serine/threonine-protein kinase Sgk3	0.731899541	0.035995056
ENSSSCT00000051906.1	ubiquitin specific peptidase 34	0.734999749	0.013450943
ENSSSCT00000019676.1	ATP synthase F0 subunit 6	0.742480403	0.033196324
ENSSSCT00000012791.3	procollagen C-endopeptidase enhancer 2	0.75345099	0.036356546
ENSSSCT00000019687.1	NADH dehydrogenase subunit 6	0.755216303	0.02782251
ENSSSCT00000019664.4	NADH dehydrogenase subunit 2	0.759444956	0.032508028
ENSSSCT00000060569.1	transmembrane protein 230	0.764695683	0.019516445
ENSSSCT00000043338.1	X-ray repair cross complementing 4	0.767920632	0.048837809
ENSSSCT00000066360.1	FK506 binding protein 5	0.783590163	0.045552846
ENSSSCT00000019689.3	cytochrome b	0.792168542	0.033234044
ENSSSCT00000019682.3	NADH dehydrogenase subunit 4	0.794780371	0.0283062
ENSSSCT00000042533.1	UDP-glucuronosyltransferase 2C1-like	0.82704293	0.039496397
ENSSSCT00000029057.2	glycoprotein, alpha-galactosyltransferase 1 pseudogene	0.830476287	0.049575848
ENSSSCT00000061719.1	SEC24 homolog A, COPII coat complex component	0.831943709	0.015819541
ENSSSCT00000019686.3	NADH dehydrogenase subunit 5	0.833613737	0.037175341
ENSSSCT00000019681.2	NADH dehydrogenase subunit 4L	0.857353101	0.031726774
ENSSSCT00000014592.3	SPT2 chromatin protein domain containing 1	0.859952181	0.046137277
ENSSSCT00000056248.1	surfactant associated 2	0.861459608	0.045124686
ENSSSCT00000018461.3	erbb2 interacting protein	0.868735176	0.036093288
ENSSSCT00000035892.2	TatD DNase domain containing 1	0.88067262	0.004375695
ENSSSCT00000016727.3	scinderin	0.90024785	0.047735547

ENSSSCT00000005070.3	ring finger protein 111	0.904421857	0.034585423
ENSSSCT00000001791.3	nuclear transcription factor Y subunit alpha	0.9389861	0.049653044
ENSSSCT00000058080.1	calpastatin	0.939436745	0.003721882
ENSSSCT00000025069.2	limb and CNS expressed 1	0.947359547	0.036153737
ENSSSCT00000066118.1	matrin 3	0.969395536	0.041862259
ENSSSCT00000057295.1	TATA-box binding protein associated factor, RNA polymerase I subunit A	0.980510138	0.039378275
ENSSSCT00000059541.1	NUMB, endocytic adaptor protein	0.994074682	0.039717082
ENSSSCT00000056975.1	dynein cytoplasmic 2 light intermediate chain 1	1.026590368	0.027895702
ENSSSCT00000049279.1	N/A	1.046984995	0.022889628
ENSSSCT00000019506.4	arachidonate 12-lipoxygenase, 12S type	1.119344876	0.024131485
ENSSSCT00000046065.1	high mobility group box 1	1.141810055	0.030791976
ENSSSCT00000058817.1	gap junction protein beta 3	1.213171597	0.044337761
ENSSSCT00000003507.3	lin-7 homolog B, crumbs cell polarity complex component	1.254493799	0.033275062
ENSSSCT00000013984.4	renin binding protein	1.319414914	0.016990343
ENSSSCT00000034393.2	nuclear factor of activated T cells 2	1.396573343	0.034885175
ENSSSCT00000033983.2	integrator complex subunit 6 like	1.422027797	0.042459301
ENSSSCT00000039888.1	CD47 molecule	1.434350484	0.007193526
ENSSSCT00000042063.1	hydroxysteroid dehydrogenase like 1	1.446927744	0.044418604
ENSSSCT00000065528.1	tRNA splicing endonuclease subunit 34	1.529526473	0.030499766
ENSSSCT00000059149.1	ADP ribosylation factor interacting protein 1	1.565048883	0.044994446
ENSSSCT00000057278.1	solute carrier family 30 member 5	1.571714421	0.005059359
ENSSSCT00000000313.3	zinc finger protein 385A	1.607845139	0.049211061
ENSSSCT00000035470.2	motile sperm domain containing 1	1.619021758	0.014229088
ENSSSCT00000059338.1	N/A	1.625621682	0.001694636
ENSSSCT00000059636.1	DnaJ heat shock protein family (Hsp40) member C24	1.659996701	0.035972141
ENSSSCT00000013101.3	collagen type VIII alpha 1 chain	1.661266493	0.011333413
ENSSSCT00000047450.1	ClpB homolog, mitochondrial AAA ATPase chaperonin	1.692150038	0.024425479
ENSSSCT00000017314.3	growth factor receptor bound protein 14	1.72291377	0.024884055
ENSSSCT00000054316.1	myosin heavy chain 11	1.72472002	0.049084891
ENSSSCT00000043603.1	matrix remodeling associated 7	1.830504205	0.025622202
ENSSSCT00000054049.1	ST3 beta-galactoside alpha-2,3-sialyltransferase 6	1.882161204	0.045423658
ENSSSCT00000012961.3	tyrosine kinase non receptor 2	1.902062951	0.049726827

ENSSSCT00000045220.1	ring finger protein 219	1.954034533	0.044554292
ENSSSCT00000064461.1	kelch repeat and BTB domain containing 3	1.960119646	0.047375183
ENSSSCT00000064172.1	lunapark, ER junction formation factor	1.997218285	0.033387752
ENSSSCT00000032954.2	interleukin 33	2.002789152	0.033050892
ENSSSCT00000028858.2	apoptotic peptidase activating factor 1	2.040512981	0.003999006
ENSSSCT00000023681.2	complement C3	2.069494032	0.002517851
ENSSSCT00000038072.1	polyhomeotic homolog 3	2.077968221	0.040782502
ENSSSCT00000051632.1	pre-mRNA processing factor 6	2.161780974	0.021525416
ENSSSCT00000010807.3	tescalcin	2.164993187	0.040849618
ENSSSCT00000065122.1	mitochondrial calcium uptake 1	2.23415028	0.026707955
ENSSSCT00000038407.1	ATPase family, AAA domain containing 5	2.259818377	0.037806055
ENSSSCT00000017668.3	phenylalanyl-tRNA synthetase subunit beta	2.272078767	0.039646535
ENSSSCT00000033380.2	Fc fragment of IgA and IgM receptor	2.35366181	0.030279916
ENSSSCT00000000448.3	retinol dehydrogenase 16 (all-trans)	2.399917181	0.020413242
ENSSSCT00000040161.1	regulatory factor X7	2.414353755	0.038800505
ENSSSCT00000063614.1	oviductal glycoprotein 1	2.442069345	0.031603792
ENSSSCT00000013823.3	stromal antigen 2	2.443585152	0.01581054
ENSSSCT00000049905.1	cysteine rich protein 3	2.459211083	0.008013765
ENSSSCT00000038494.1	zinc finger protein 22	2.463597258	0.010806034
ENSSSCT00000039185.1	kelch like family member 24	2.481161163	0.022801934
ENSSSCT00000051840.1	cytoplasmic linker associated protein 2	2.516621907	0.028354365
ENSSSCT00000038930.1	O-GlcNAcase	2.561542887	0.024833491
ENSSSCT00000054336.1	prune exopolyphosphatase 1	2.570061775	0.024742052
ENSSSCT00000043726.1	centrosomal protein 164	2.645271879	0.04895139
ENSSSCT00000065697.1	SREBF chaperone	2.70826019	0.007401059
ENSSSCT00000049855.1	solute carrier family 38 member 6	2.726421909	0.005895477
ENSSSCT00000054259.1		2.778472624	0.041750346
ENSSSCT00000054879.1	deoxyribose-phosphate aldolase	2.784386821	0.005083522
ENSSSCT00000007741.3	N/A	2.926480326	0.002353708
ENSSSCT00000040830.1	lactate dehydrogenase A	2.959469677	0.011738444
ENSSSCT00000001147.3	TBC1 domain family member 7	3.006870677	0.003556076
ENSSSCT00000062599.1	phospholipase C beta 4	3.017118093	0.024254038
ENSSSCT00000000873.3	anoctamin 6	3.033938674	0.04161469

ENSSSCT00000060910.1	transmembrane 7 superfamily member 3	3.333125425	0.019481025
ENSSSCT00000050336.1	N/A	3.429917188	0.017613485
ENSSSCT00000040036.1	nuclear transcription factor Y subunit gamma	3.69865234	0.022031301
ENSSSCT00000029478.2	formin binding protein 4	3.985865578	0.000388679
ENSSSCT00000063099.1	N/A	5.493951298	0.000538038

Supplementary Table S3. Differentially expressed genes in Zn100 group vs LQ100 group

Transcript ID	Gene description	Log2 fold change	*p*-value
ENSSSCT00000003882.3	phospholipase A2 group IIA	-24.18392058	4.27E-16
ENSSSCT00000063073.1	N/A	-5.903916273	0.000112334
ENSSSCT00000053941.1	N/A	-5.273874536	0.048456491
ENSSSCT00000050495.1	N/A	-5.014812688	0.004904666
ENSSSCT00000034766.2	ribosomal protein S16	-4.611898167	0.011524424
ENSSSCT00000041456.1	N/A	-4.577389615	0.044041698
ENSSSCT00000051287.1	heterochromatin protein 1 binding protein 3	-4.183412477	0.002541254
ENSSSCT00000066245.1	ribosomal protein L19	-3.78767304	0.006272101
ENSSSCT00000055308.1	phosphatidylinositol glycan anchor biosynthesis class X	-3.527025558	0.010508811
ENSSSCT00000054526.1	N-glycanase 1	-3.304585282	0.002805301
ENSSSCT00000044293.1	joining chain of multimeric IgA and IgM	-3.278880633	0.002987103
ENSSSCT00000002379.3	chromodomain helicase DNA binding protein 8	-3.258788529	0.008217896
ENSSSCT00000018034.3	ubiquitin conjugating enzyme E2 H	-3.159968593	0.002541668
ENSSSCT00000029955.2	phosphofructokinase, muscle	-3.155249834	0.034885607
ENSSSCT00000053191.1	N/A	-3.141907636	0.002868669
ENSSSCT00000029449.2	nucleolar protein interacting with the FHA domain of MKI67	-3.118066083	2.25E-05
ENSSSCT00000038131.1	nuclear transcription factor Y subunit gamma	-3.079956696	0.003515179
ENSSSCT00000059280.1	spectrin alpha, non-erythrocytic 1	-2.884628886	0.00805423
ENSSSCT00000039720.1	sulfatase modifying factor 1	-2.844852009	0.039321202
ENSSSCT00000040161.1	regulatory factor X7	-2.833137172	0.00908819
ENSSSCT00000016994.3	integrator complex subunit 7	-2.728006311	0.022725629
ENSSSCT00000058306.1	basic leucine zipper and W2 domains 2	-2.712461916	0.00713979
ENSSSCT00000061087.1	DNA methyltransferase 3 alpha	-2.622227139	0.036543353
ENSSSCT00000037570.1	protein tyrosine phosphatase, receptor type F	-2.62108055	0.003885315
ENSSSCT00000002344.2	N/A	-2.593953515	0.002324262
ENSSSCT00000059451.1	basic helix-loop-helix family member b9	-2.531244356	0.019709583
ENSSSCT00000049089.1	ITPRIP like 1	-2.530826135	0.04405131
ENSSSCT00000061972.1	RB binding protein 4, chromatin remodeling factor	-2.46768638	0.006887848

ENSSSCT00000057440.1	shisa family member 5	-2.453398039	0.005664222
ENSSSCT00000031253.2	oxidation resistance 1	-2.433108324	0.009657193
ENSSSCT00000062108.1	prickle planar cell polarity protein 4	-2.402351002	0.015757073
ENSSSCT00000005458.3	zwilch kinetochore protein	-2.386681586	0.022207086
ENSSSCT00000039327.1	selenium binding protein 1	-2.379572571	0.038065514
ENSSSCT00000057380.1	transmembrane protein 51	-2.368501555	0.039642755
ENSSSCT00000040326.1	chromosome 11 open reading frame 74	-2.365644781	0.038045998
ENSSSCT00000012564.3	chromosome 3 open reading frame 67	-2.353099185	0.03930438
ENSSSCT00000025172.2	bactericidal permeability increasing protein	-2.331708999	0.014941106
ENSSSCT00000049559.1	N/A	-2.314749201	0.02657465
ENSSSCT00000050316.1	HMG-box containing 4	-2.290427612	0.016011168
ENSSSCT00000060710.1	protein phosphatase 4 regulatory subunit 3B	-2.261911416	0.027099403
ENSSSCT00000009621.3	ras homolog family member H	-2.250374708	0.022657354
ENSSSCT00000046817.1	tyrosine 3-monooxygenase/tryptophan 5-monooxygenase activation protein zeta	-2.247118058	0.015901325
ENSSSCT00000058789.1	ArfGAP with FG repeats 1	-2.242777923	0.022847104
ENSSSCT00000009044.3	lithostathine-like	-2.241301125	0.023877355
ENSSSCT00000025743.2	chromosome 5 open reading frame 30	-2.2306236	0.029331195
ENSSSCT00000049484.1	S100 calcium binding protein G	-2.20213433	0.023640143
ENSSSCT00000055051.1	Ras association domain family member 1	-2.181828543	0.0136707
ENSSSCT00000042792.1	phosphatidylinositol transfer protein membrane associated 2	-2.140741074	0.031567014
ENSSSCT00000057213.1	forkhead box N2	-2.129238986	0.024263923
ENSSSCT00000043897.1	fatty acyl-CoA reductase 1	-2.10787322	0.005684661
ENSSSCT00000040591.1	zinc finger protein 280D	-2.092444188	0.042463155
ENSSSCT00000045691.1		-2.024328848	0.049249475
ENSSSCT00000054734.1	SWI/SNF-related, matrix-associated actin-dependent regulator of chromatin, subfamily a, containing DEAD/H box 1	-2.012089904	0.041471078
ENSSSCT00000010484.3	transmembrane and coiled-coil domains 3	-2.000529861	0.035595559
ENSSSCT00000011794.3	synaptonemal complex central element protein 1	-1.981172525	0.049690259
ENSSSCT00000046440.1	tetraspanin 5	-1.929212169	0.007939269
ENSSSCT00000055045.1	TBC1 domain family member 7	-1.923133113	0.047455512

ENSSSCT00000050638.1	HtrA serine peptidase 4	-1.869959914	0.046233965
ENSSSCT00000050197.1	ITPR interacting domain containing 2	-1.823205528	0.043321334
ENSSSCT00000056966.1	N/A	-1.7790778	0.034539726
ENSSSCT00000063561.1	chromosome transmission fidelity factor 8	-1.757218674	0.04724822
ENSSSCT00000029842.2	solute carrier family 40 member 1	-1.737232877	0.011947468
ENSSSCT00000057915.1	retinoic acid receptor responder 2	-1.723709625	0.027064178
ENSSSCT00000009007.3	pentatricopeptide repeat domain 3	-1.712602524	0.017737748
ENSSSCT00000048045.1	phosphatidylinositol 3-kinase catalytic subunit type 3	-1.667678559	0.017049353
ENSSSCT00000057354.1	EF-hand calcium binding domain 14	-1.649656928	0.031930266
ENSSSCT00000010929.3	rhomboid domain containing 3	-1.626530265	0.022042211
ENSSSCT00000047652.1	kinesin family member 2C	-1.624579843	0.022274968
ENSSSCT00000063644.1	solute carrier family 6 member 9	-1.615778965	0.036602397
ENSSSCT00000032434.2	low density lipoprotein receptor class A domain containing 4	-1.615542066	0.036176792
ENSSSCT00000004712.3	zinc finger containing ubiquitin peptidase 1	-1.589657178	0.041064465
ENSSSCT00000050142.1	ER degradation enhancing alpha-mannosidase like protein 3	-1.587206326	0.031895276
ENSSSCT00000059923.1	F-box protein 25	-1.567997359	0.003585989
ENSSSCT00000037953.1	thyroid hormone receptor interactor 12	-1.538103572	0.019509486
ENSSSCT00000049618.1	thyroid hormone receptor alpha	-1.534849003	0.037602552
ENSSSCT00000059894.1	NOP58 ribonucleoprotein	-1.479593493	0.012833619
ENSSSCT00000005130.3	GA binding protein transcription factor subunit beta 1	-1.471669624	0.025680311
ENSSSCT00000005185.3	WD repeat domain 76	-1.45035634	0.02280728
ENSSSCT00000057182.1	TNF receptor superfamily member 4	-1.449810613	0.014566984
ENSSSCT00000005112.3	LysM domain containing 2	-1.445966265	0.04268515
ENSSSCT00000065335.1	O-6-methylguanine-DNA methyltransferase	-1.445292163	0.039976214
ENSSSCT00000044950.1	N/A	-1.406538552	0.034612061
ENSSSCT00000061913.1	neuronal regeneration related protein	-1.294440249	0.00319752
ENSSSCT00000066361.1	ribosomal protein L15	-1.276455575	0.018411358
ENSSSCT00000008849.3	inositol-pentakisphosphate 2-kinase	-1.274053208	0.035893952
ENSSSCT00000010386.3	MYC binding protein 2, E3 ubiquitin protein ligase	-1.269447142	0.025478388
ENSSSCT00000034359.2	CD27 molecule	-1.181666162	0.034698578

ENSSSCT00000061741.1	CCR4-NOT transcription complex subunit 2	-1.06970357	0.021297216
ENSSSCT00000041061.1	dual specificity tyrosine phosphorylation regulated kinase 1A	-1.058960738	0.006764951
ENSSSCT00000000767.2	CD27 molecule	-1.056946607	0.00259463
ENSSSCT00000064264.1	mitochondrial transcription termination factor 1	-1.055870412	0.018975659
ENSSSCT00000001560.3	euchromatic histone lysine methyltransferase 2	-1.052596992	0.022855879
ENSSSCT00000041049.1	tetraspanin 5	-1.045469275	0.0461556
ENSSSCT00000058408.1	splicing factor 3b subunit 1	-1.023311139	0.030200152
ENSSSCT00000050784.1	FA complementation group D2	-1.000350595	0.028431835
ENSSSCT00000053964.1	importin 11	-0.955712799	0.039516324
ENSSSCT00000060206.1	SURF1, cytochrome c oxidase assembly factor	-0.940829476	0.008521679
ENSSSCT00000036436.2	cytotoxic T-lymphocyte associated protein 4	-0.910269023	0.028601332
ENSSSCT00000057503.1	cold inducible RNA binding protein	-0.863268945	0.035670232
ENSSSCT00000044338.1	mitogen-activated protein kinase kinase kinase kinase 4	-0.845322835	0.038491883
ENSSSCT00000022399.2	lysine rich nucleolar protein 1	-0.824858714	0.043510511
ENSSSCT00000040898.1	microtubule-actin crosslinking factor 1	-0.798109417	0.01934093
ENSSSCT00000045972.1	phospholipase D family member 3	-0.76736448	0.037087695
ENSSSCT00000040645.1	interferon regulatory factor 8	-0.748837959	0.030383756
ENSSSCT00000029013.2	RNA binding motif protein 18	-0.734450802	0.025033149
ENSSSCT00000052923.1	nuclear body protein SP140-like protein	-0.715121689	0.010903301
ENSSSCT00000048725.1	T cell receptor beta variable 30 (gene/pseudogene)	-0.704786566	0.004083085
ENSSSCT00000018767.3	tweety family member 2	-0.697099384	0.034027196
ENSSSCT00000035758.2	ubiquitin protein ligase E3 component n-recognin 5	-0.687676323	0.037806198
ENSSSCT00000008081.3	translocase of outer mitochondrial membrane 34	-0.670746509	0.042075773
ENSSSCT00000014304.3	cytochrome b561 family member A3	-0.647289313	0.00881016
ENSSSCT00000030066.2	adenosine deaminase	-0.646331474	0.022868938
ENSSSCT00000063738.1	transcription factor 3	-0.638789162	0.023109493
ENSSSCT00000042072.1	TBC1 domain family member 10C	-0.633671941	0.030256857
ENSSSCT00000018469.3	RAD17 checkpoint clamp loader component	-0.631047387	0.016859362
ENSSSCT00000055932.1	transmembrane protein 11	-0.625290644	0.02158401

ENSSSCT00000052940.1	CCR4-NOT transcription complex subunit 8	-0.619616656	0.010886306
ENSSSCT00000000750.3	lymphocyte activating 3	-0.618504424	0.049781452
ENSSSCT00000043658.1	tubulin beta 4B class IVb	-0.604107781	0.011313731
ENSSSCT00000017944.3	N/A	-0.59435051	0.004235215
ENSSSCT00000060863.1	SMU1, DNA replication regulator and spliceosomal factor	-0.589077657	0.019440256
ENSSSCT00000017010.3	hydroxysteroid 11-beta dehydrogenase 1	-0.586769508	0.048920186
ENSSSCT00000003223.2	hematopoietic cell signal transducer	-0.566119264	0.034943612
ENSSSCT00000043336.1	copine 1	-0.56097322	0.047978114
ENSSSCT00000063466.1	lipin 2	-0.546998052	0.014170445
ENSSSCT00000065676.1	fibrillarin	-0.519712132	0.047613933
ENSSSCT00000008412.3	POP7 homolog, ribonuclease P/MRP subunit	-0.515692885	0.03190433
ENSSSCT00000050664.1	proteasome assembly chaperone 3	-0.511376159	0.039106159
ENSSSCT00000018224.3	myosin IG	-0.504672345	0.048549555
ENSSSCT00000037720.1	N/A	-0.497360667	0.037542644
ENSSSCT00000048683.1	GTPase, IMAP family member 6	-0.495157949	0.009528821
ENSSSCT00000012625.3	ADP ribosylation factor like GTPase 8B	-0.488532357	0.031238897
ENSSSCT00000043450.1	eukaryotic translation initiation factor 3 subunit K	-0.46669003	0.00413691
ENSSSCT00000008748.3	mitochondrial ribosomal protein L28	-0.461708479	0.010068575
ENSSSCT00000054928.1	eukaryotic translation elongation factor 1 alpha 1	-0.439887378	0.031252146
ENSSSCT00000048609.1	FCF1, rRNA-processing protein	-0.437454204	0.004550425
ENSSSCT00000054538.1	ribosomal protein S9	-0.432725296	0.018322695
ENSSSCT00000036365.2	ubiquitously expressed prefoldin like chaperone	-0.419394093	0.007283995
ENSSSCT00000028688.2	SERTA domain containing 1	-0.41703655	0.037230804
ENSSSCT00000039677.1	ribosomal protein L28	-0.413770633	0.028878982
ENSSSCT00000045113.1	selenoprotein W	-0.409936529	0.002341962
ENSSSCT00000062407.1	SUZ RNA binding domain containing 1	-0.403216376	0.019853827
ENSSSCT00000038751.1	metallo-beta-lactamase domain containing 1	-0.400736821	0.030389926
ENSSSCT00000049267.1	eukaryotic translation initiation factor 3 subunit K	-0.39797562	0.007682182
ENSSSCT00000046389.1	GrpE like 1, mitochondrial	-0.396359027	0.00107458
ENSSSCT00000044740.1	transmembrane protein 273	-0.393323603	0.025017148
ENSSSCT00000044715.1	glutaredoxin 3	-0.391033313	0.020628526

ENSSSCT00000048452.1	ribosomal protein L28	-0.382396779	0.039971914
ENSSSCT00000052185.1	cathepsin D	-0.379429826	0.023339239
ENSSSCT00000059106.1	RNA binding motif protein 4	-0.37801962	0.045763209
ENSSSCT00000048624.1	ring finger protein 113A	-0.377197345	0.032782835
ENSSSCT00000061168.1	ribosomal protein S2	-0.366684797	0.039870068
ENSSSCT00000012340.3	ribosomal protein L14	-0.361383902	0.00031845
ENSSSCT00000029505.2	PX domain containing 1	-0.356353668	0.03478196
ENSSSCT00000056491.1	ribosomal protein L4	-0.351119746	0.015056566
ENSSSCT00000014242.3	tRNA methyltransferase subunit 11-2	-0.348120015	0.009094574
ENSSSCT00000064200.1	ribosomal protein L19	-0.347747544	0.015574285
ENSSSCT00000048373.1	eukaryotic translation initiation factor 5A	-0.346990551	0.015380492
ENSSSCT00000054388.1	BRCA2 and CDKN1A interacting protein	-0.346914607	0.033746112
ENSSSCT00000045474.1	ribosomal protein L10a	-0.346860291	0.001255086
ENSSSCT00000064349.1	RNA polymerase II subunit D	-0.345325627	0.030774803
ENSSSCT00000048468.1	solute carrier family 25 member 6	-0.341890138	0.008967768
ENSSSCT00000025029.2	NOP53 ribosome biogenesis factor	-0.33552327	0.006326654
ENSSSCT00000014851.3	ribosomal protein S28	-0.333313861	0.006253158
ENSSSCT00000059130.1	nuclear body protein SP140-like protein	-0.332860853	0.035318268
ENSSSCT00000059836.1	small nuclear ribonucleoprotein D2 polypeptide	-0.332169712	0.049566313
ENSSSCT00000061274.1	NADH:ubiquinone oxidoreductase complex assembly factor 8	-0.330542747	0.046710607
ENSSSCT00000028682.2	ribosomal protein L13	-0.328913177	0.017097963
ENSSSCT00000031015.3	ribosomal protein L8	-0.327301379	0.027341663
ENSSSCT00000064812.1	actin related protein 2/3 complex subunit 4	-0.327266631	0.042246532
ENSSSCT00000001675.3	ribosomal protein S18	-0.322883333	0.008225656
ENSSSCT00000044425.1	dihydrouridine synthase 1 like	-0.319153636	0.04986722
ENSSSCT00000024892.2	ribosomal protein S2	-0.315605395	0.028723504
ENSSSCT00000044605.1	hydroxysteroid 11-beta dehydrogenase 1 like	-0.315134803	0.041812024
ENSSSCT00000063010.1	ribosomal protein L3	-0.311062472	0.003414329
ENSSSCT00000004003.3	LCK proto-oncogene, Src family tyrosine kinase	-0.309201863	0.027082817
ENSSSCT00000044760.1	ribosomal protein L32	-0.308745926	0.018209917
ENSSSCT00000003515.3	ribosomal protein S11	-0.308050616	0.045496793
ENSSSCT00000014280.3	eukaryotic translation elongation factor 1 gamma	-0.306982518	0.010288993
ENSSSCT00000016207.3	ribosomal protein S3	-0.303561107	0.00178912

ENSSSCT00000007934.3	COMM domain containing 7	-0.303292416	0.041918507
ENSSSCT00000003287.3	eukaryotic translation initiation factor 3 subunit K	-0.301831875	0.008129704
ENSSSCT00000004960.3	eukaryotic translation elongation factor 1 alpha 1	-0.301012103	0.001097065
ENSSSCT00000049763.1	ribosomal protein lateral stalk subunit P0	-0.29006573	0.006427998
ENSSSCT00000045427.1	anaphase promoting complex subunit 11	-0.285807903	0.044409636
ENSSSCT00000029345.2	UBX domain protein 1	-0.280989372	0.009365786
ENSSSCT00000014037.3	ribosomal protein lateral stalk subunit P2	-0.278845121	0.023961817
ENSSSCT00000041942.1	ribosomal protein S19	-0.273889547	0.03719649
ENSSSCT00000015437.3	ribosomal protein S23	-0.27225382	0.045699038
ENSSSCT00000066267.1	U2 small nuclear RNA auxiliary factor 1	-0.268879344	0.017634077
ENSSSCT00000014925.3	eukaryotic translation initiation factor 3 subunit G	-0.265657041	0.030376463
ENSSSCT00000040918.1	ribosomal protein L5	-0.265597496	0.029521428
ENSSSCT00000001720.3	ribosomal protein L10a	-0.26249581	0.03248726
ENSSSCT00000052787.1	eukaryotic translation elongation factor 1 delta	-0.26018951	0.012740554
ENSSSCT00000018679.3	eukaryotic translation initiation factor 4A3	-0.253388464	0.048564297
ENSSSCT00000005457.3	ribosomal protein L4	-0.252519378	0.015123591
ENSSSCT00000061738.1	ribosomal protein L18	-0.252467063	0.044815468
ENSSSCT00000008725.3	small nuclear ribonucleoprotein U11/U12 subunit 25	-0.247774692	0.023367155
ENSSSCT00000027316.2	ribosomal protein L29	-0.245583248	0.025797705
ENSSSCT00000014615.3	ribosomal protein S13	-0.242742242	0.022929899
ENSSSCT00000012642.3	actin related protein 2/3 complex subunit 4	-0.237900618	0.044666642
ENSSSCT00000016669.3	sorcin	-0.235067559	0.03085186
ENSSSCT00000013078.3	ribosomal protein L24	-0.229063233	0.004057949
ENSSSCT00000015919.3	ribosomal protein L27a	-0.222205158	0.018227443
ENSSSCT00000015001.3	telomerase RNA component interacting RNase	-0.2140207	0.049415263
ENSSSCT00000043834.1	ribosomal protein S20	-0.213240738	0.009725433
ENSSSCT00000006622.3	eukaryotic translation initiation factor 3 subunit E	-0.210572087	0.04223635
ENSSSCT00000011019.3	small nuclear ribonucleoprotein D3 polypeptide	-0.205395083	0.035395387

ENSSSCT00000065049.1	FAU, ubiquitin like and ribosomal protein S30 fusion	-0.201332495	0.027209036
ENSSSCT00000024076.2	ribosomal protein L26	-0.19982986	0.049854546
ENSSSCT00000006146.3	proteasome subunit beta 7	-0.186225711	0.043890188
ENSSSCT00000046697.1	eukaryotic translation initiation factor 3 subunit H	-0.160579682	0.045221272
ENSSSCT00000000090.2	activating transcription factor 4	-0.15098453	0.046893499
ENSSSCT00000017252.3	glutathione-disulfide reductase	0.228069039	0.043028459
ENSSSCT00000000975.3	leukotriene A4 hydrolase	0.249415173	0.039710764
ENSSSCT00000026750.2	ZFP36 ring finger protein	0.249767753	0.048166915
ENSSSCT00000018376.3	3-hydroxy-3-methylglutaryl-CoA synthase 1	0.273484968	0.01957876
ENSSSCT00000053892.1	ADP ribosylation factor 1	0.301933511	0.026476638
ENSSSCT00000024166.2	farnesyl-diphosphate farnesyltransferase 1	0.302054013	0.020718497
ENSSSCT00000062663.1	malic enzyme 2	0.34409655	0.042623887
ENSSSCT00000056298.1	basic helix-loop-helix family member e40	0.383593617	0.019060614
ENSSSCT00000003473.3	KDEL endoplasmic reticulum protein retention receptor 1	0.386555748	0.044682477
ENSSSCT00000056665.1	parvin alpha	0.390615534	0.048743121
ENSSSCT00000011350.3	glutamate dehydrogenase 1	0.392163974	0.005815942
ENSSSCT00000018974.2	junction plakoglobin	0.409097285	0.041590549
ENSSSCT00000042242.1	tumor protein D52	0.42286583	0.021536879
ENSSSCT00000045246.1	SH3 domain binding glutamate rich protein like 2	0.423793266	0.037406043
ENSSSCT00000053997.1	RAS like proto-oncogene B	0.440418268	0.006508252
ENSSSCT00000056807.1	MAX dimerization protein 1	0.442945948	0.044731384
ENSSSCT00000056464.1	dual specificity phosphatase 5	0.44821078	0.036577045
ENSSSCT00000050201.1	acyl-CoA oxidase 1	0.45740727	0.022480224
ENSSSCT00000016766.2	carnitine O-octanoyltransferase	0.462896089	0.047429214
ENSSSCT00000004193.3	cystathionine gamma-lyase	0.469824567	0.026811527
ENSSSCT00000009288.3	galactose mutarotase	0.470936308	0.014467425
ENSSSCT00000041118.1	protein kinase C delta	0.474101922	0.049889822
ENSSSCT00000007115.3	lamin A/C	0.498710376	0.026724367
ENSSSCT00000017880.3	insulin induced gene 1	0.499147213	0.010739238
ENSSSCT00000041873.1	zinc finger and BTB domain containing 7B	0.50388769	0.042467941
ENSSSCT00000052265.1	erythrocyte membrane protein band 4.1 like 3	0.505882467	0.04957682
ENSSSCT00000027607.2	aldehyde dehydrogenase 1 family member A1	0.506628256	0.011325221

ENSSSCT00000044681.1	interleukin 13 receptor subunit alpha 1	0.52215864	0.017111338
ENSSSCT00000055492.1	filamin binding LIM protein 1	0.52598246	0.043158847
ENSSSCT00000017060.3	complement component 4 binding protein, alpha	0.534284994	0.012598024
ENSSSCT00000012958.3	solute carrier family 51 alpha subunit	0.543047004	0.012869432
ENSSSCT00000043928.1	N/A	0.546006946	0.029306386
ENSSSCT00000057200.1	N/A	0.564015399	0.023775601
ENSSSCT00000007531.3	sorting nexin 7	0.564521597	0.046556275
ENSSSCT00000058742.1	enoyl-CoA hydratase and 3-hydroxyacyl CoA dehydrogenase	0.566368513	0.049208472
ENSSSCT00000026717.2	anterior gradient 2, protein disulphide isomerase family member	0.5687442	0.04719697
ENSSSCT00000061552.1	SOS Ras/Rac guanine nucleotide exchange factor 1	0.577627062	0.015410144
ENSSSCT00000030773.2	kelch like family member 7	0.5789791	0.046442273
ENSSSCT00000044810.1	microtubule associated scaffold protein 1	0.583234871	0.032898387
ENSSSCT00000053024.1	ras-related protein Rap-2a pseudogene	0.594844559	0.021533934
ENSSSCT00000037199.1	dual specificity phosphatase 6	0.602643778	0.009619799
ENSSSCT00000001722.3	TEA domain transcription factor 3	0.606707967	0.033378632
ENSSSCT00000031995.2	myosin heavy chain 14	0.608369334	0.022346768
ENSSSCT00000060242.1	N/A	0.622131058	0.025839191
ENSSSCT00000032098.2	family with sequence similarity 83 member E	0.626529574	0.046255128
ENSSSCT00000003075.3	hydroxysteroid 11-beta dehydrogenase 2	0.6285727	0.026553291
ENSSSCT00000062764.1	syntaxin binding protein 6	0.630177872	0.005360919
ENSSSCT00000048374.1		0.651053344	0.000459718
ENSSSCT00000040297.1	fatty acid binding protein 2	0.654992496	0.044672791
ENSSSCT00000006690.3	epithelial splicing regulatory protein 1	0.666738637	0.039224058
ENSSSCT00000041432.1	N/A	0.671084798	0.003375753
ENSSSCT00000007341.3	flavin containing monooxygenase 5	0.675439465	0.042551565
ENSSSCT00000052138.1	guanosine monophosphate reductase	0.67648381	0.029305606
ENSSSCT00000013382.4	ornithine carbamoyltransferase	0.678854595	0.019270481
ENSSSCT00000029156.2	family with sequence similarity 3 member D	0.688418873	0.021396802
ENSSSCT00000044801.1	N/A	0.699719353	0.037924239
ENSSSCT00000040699.1	phospholipase D1	0.70106968	0.035896185

ENSSSCT00000045147.1	protein phosphatase, Mg2+/Mn2+ dependent 1H	0.713428805	0.029271065
ENSSSCT00000018356.3	complement C9	0.729433761	0.027853069
ENSSSCT00000010052.3	microsomal triglyceride transfer protein	0.736458662	0.043192467
ENSSSCT00000063809.1	glycerol-3-phosphate dehydrogenase 2	0.740554029	0.028512158
ENSSSCT00000033582.2	joining chain of multimeric IgA and IgM	0.742360146	0.019224111
ENSSSCT00000035626.2	alanyl aminopeptidase, membrane	0.744203698	0.026892386
ENSSSCT00000047803.1	complement component 4 binding protein beta	0.745822862	0.021506017
ENSSSCT00000063237.1	N/A	0.751282838	0.014443562
ENSSSCT00000016224.3	tsukushi, small leucine rich proteoglycan	0.753103364	0.011264702
ENSSSCT00000029761.2	N/A	0.757557891	0.002504213
ENSSSCT00000045217.1	peripheral myelin protein 22	0.759406347	0.027215413
ENSSSCT00000066211.1	N/A	0.769239159	0.047304769
ENSSSCT00000047176.1	desmocollin 3	0.785933144	0.017273354
ENSSSCT00000027977.2	trefoil factor 2	0.788243115	0.021602008
ENSSSCT00000062230.1	tight junction protein 2	0.78872093	0.027951294
ENSSSCT00000047623.1	chloride channel accessory 1	0.796612119	0.022119187
ENSSSCT00000044005.1	N/A	0.798650358	0.0001185
ENSSSCT00000054278.1	CD46 molecule, complement regulatory protein	0.816204505	0.032528643
ENSSSCT00000056923.1	N/A	0.819251357	0.017834667
ENSSSCT00000047974.1	interferon induced protein 44	0.831182492	0.036912316
ENSSSCT00000063551.1	N/A	0.832479647	0.0281713
ENSSSCT00000045881.1	chloride intracellular channel 5	0.83933671	0.02949258
ENSSSCT00000052141.1	solute carrier family 5 member 1	0.842309411	0.037201226
ENSSSCT00000055736.1	acyl-CoA synthetase long chain family member 3	0.857990312	0.04818076
ENSSSCT00000065219.1	tubulin polymerization promoting protein	0.899671484	0.042575931
ENSSSCT00000039153.1	N/A	0.900723239	0.044898055
ENSSSCT00000046834.1	four and a half LIM domains 2	0.902361244	0.017710037
ENSSSCT00000003832.3	podoplanin	0.902714001	0.012745291
ENSSSCT00000000447.3	myosin IA	0.903183814	0.029308371
ENSSSCT00000057491.1	joining chain of multimeric IgA and IgM	0.919984466	0.000248301
ENSSSCT00000027766.2	Fc fragment of IgG binding protein	0.924706741	0.003238298

ENSSSCT00000002650.3	Fos proto-oncogene, AP-1 transcription factor subunit	0.944337622	0.000745439
ENSSSCT00000047537.1	Fc fragment of IgG binding protein	0.959046505	0.034806463
ENSSSCT00000060871.1	joining chain of multimeric IgA and IgM	0.995406747	0.000104136
ENSSSCT00000049254.1	nudE neurodevelopment protein 1 like 1	1.006770611	0.046749833
ENSSSCT00000064583.1	N/A	1.019924379	0.012900683
ENSSSCT00000058969.1	guanosine monophosphate reductase	1.020975407	0.036952948
ENSSSCT00000047658.1	ubiquitin protein ligase E3 component n-recognin 2	1.021059766	0.048499307
ENSSSCT00000055231.1	N/A	1.039972458	0.045817247
ENSSSCT00000059114.1	spermatogenesis associated 2	1.042826532	0.035361058
ENSSSCT00000040369.1	sorbin and SH3 domain containing 2	1.091974767	0.048312663
ENSSSCT00000040290.1	N/A	1.127059856	0.0151807
ENSSSCT00000059021.1	UPF2, regulator of nonsense mediated mRNA decay	1.190386416	0.02916305
ENSSSCT00000044637.1	carboxypeptidase O	1.198948792	0.010033733
ENSSSCT00000043799.1	N/A	1.203042507	0.01904114
ENSSSCT00000065387.1	erythrocyte membrane protein band 4.1 like 2	1.206953817	0.033360419
ENSSSCT00000052491.1	bactericidal permeability increasing protein	1.229461355	0.010903429
ENSSSCT00000045493.1	ubiquitin specific peptidase 32	1.251892925	0.02423592
ENSSSCT00000058777.1	F-box protein 25	1.253067087	0.006058841
ENSSSCT00000014625.3	cytochrome P450 family 2 subfamily R member 1	1.277134606	0.011235581
ENSSSCT00000007595.3	chloride channel accessory 1	1.298638285	0.031769943
ENSSSCT00000038671.1	solute carrier family 22 member 5	1.299272084	0.014171183
ENSSSCT00000064491.1	N/A	1.30046266	0.019382197
ENSSSCT00000052505.1	joining chain of multimeric IgA and IgM	1.30666671	0.010225436
ENSSSCT00000063710.1	N/A	1.329926051	0.005996359
ENSSSCT00000043225.1	bactericidal permeability increasing protein	1.334354418	0.018338655
ENSSSCT00000015497.3	peptidylglycine alpha-amidating monooxygenase	1.335477224	0.011811013
ENSSSCT00000053543.1	C-C motif chemokine receptor like 2	1.337946917	0.005555039
ENSSSCT00000045318.1	myosin light chain kinase	1.34489206	0.017460681
ENSSSCT00000056394.1	tetratricopeptide repeat domain 23	1.374097335	0.047170318
ENSSSCT00000042177.1	N/A	1.39697285	0.041958784

ENSSSCT00000051608.1	N/A	1.430563486	0.016759801
ENSSSCT00000056768.1	chromodomain helicase DNA binding protein 8	1.47620019	0.004074383
ENSSSCT00000055882.1	slingshot protein phosphatase 1	1.533796192	0.048694719
ENSSSCT00000010413.3	N/A	1.689959012	0.021009748
ENSSSCT00000045907.1	Rho/Rac guanine nucleotide exchange factor 2	1.745901673	0.020679246
ENSSSCT00000064141.1	DnaJ heat shock protein family (Hsp40) member C6	1.803558093	0.042643296
ENSSSCT00000050440.1	microsomal glutathione S-transferase 3	1.857675518	0.047748826
ENSSSCT00000012603.3	eukaryotic translation initiation factor 4E family member 3	1.865355431	0.040279106
ENSSSCT00000039326.1	calponin 1	1.936781763	0.016721074
ENSSSCT00000039359.1	solute carrier family 8 member A1	1.949080555	0.01633535
ENSSSCT00000018449.3	ring finger protein 180	1.989673061	0.031291852
ENSSSCT00000038274.1	biotinidase	1.997276394	0.005449842
ENSSSCT00000050120.1	chromosome 11 open reading frame 74	2.008503383	0.021134517
ENSSSCT00000059327.1	endoplasmic reticulum to nucleus signaling 2	2.048283023	0.045395869
ENSSSCT00000064256.1	Rho/Rac guanine nucleotide exchange factor 2	2.057061738	0.041736847
ENSSSCT00000050656.1	kizuna centrosomal protein	2.181235801	0.028981269
ENSSSCT00000025055.2	aquaporin 8	2.206333179	0.046091514
ENSSSCT00000044500.1	cordon-bleu WH2 repeat protein like 1	2.232628197	0.037869611
ENSSSCT00000066304.1	zinc finger FYVE-type containing 1	2.258667338	0.03915558
ENSSSCT00000047067.1	thyroid hormone receptor alpha	2.285011402	0.002745122
ENSSSCT00000054330.1	transmembrane protein 150A	2.354042851	0.020546322
ENSSSCT00000004585.3	NHS like 1	2.439189384	0.014560763
ENSSSCT00000053777.1	ATPase phospholipid transporting 11B (putative)	2.464333431	0.030334722
ENSSSCT00000044688.1	OCIA domain containing 2	2.473570602	0.04668636
ENSSSCT00000048631.1	B cell CLL/lymphoma 9	2.486256172	0.033295044
ENSSSCT00000047493.1	nodal modulator 1	2.520530438	0.011909324
ENSSSCT00000066110.1	sperm antigen with calponin homology and coiled-coil domains 1	2.560546363	0.012158755
ENSSSCT00000032648.2	midline 1	2.784179005	0.006974374
ENSSSCT00000033983.2	integrator complex subunit 6 like	2.907261453	0.009317518
ENSSSCT00000031974.2	metallothionein-1E-like	3.570081257	0.039755088
ENSSSCT00000041081.1	N/A	3.575680262	0.021999837

ENSSSCT00000030537.2	histone H4	3.649738452	0.027355311
ENSSSCT00000065870.1	N/A	3.791101458	0.00720061
ENSSSCT00000043706.1	phosphofructokinase, muscle	3.891504668	0.003391823
ENSSSCT00000039549.1	tetraspanin 5	3.906028911	0.005199685
ENSSSCT00000009797.3	solute carrier family 4 member 4	4.03921352	0.014906521
ENSSSCT00000044624.1	N/A	4.273336318	0.00280932
ENSSSCT00000063767.1	N/A	4.820942794	0.031046776

Supplementary Table S4. Genes related to transporter activity

ID	Log2 fold change	*p*-value	Gene Name	Panther Family
**Zn50 vs LQ50**				
ENSSSCT00000 010090	-3.2878	0.0012	Uncharacterized protein;LOC110255210 ;ortholog	ATP-BINDING CASSETTE SUB-FAMILY G MEMBER 2 (PTHR19241:SF311)
ENSSSCT00000 055096	-2.8582	0.0244	XK-related protein;XKR9;ortholog	XK-RELATED PROTEIN 9 (PTHR16024:SF13)
ENSSSCT00000 037785	-2.1417	0.0210	Sortilin related VPS10 domain containing receptor 2;SORCS2;ortholog	VPS10 DOMAIN-CONTAINING RECEPTOR SORCS2 (PTHR12106:SF9)
ENSSSCT00000 054913	-1.9534	0.0041	Solute carrier family 12 member 6;SLC12A6;ortholog	SOLUTE CARRIER FAMILY 12 MEMBER 6 (PTHR11827:SF66)
ENSSSCT00000 061852	-1.6591	0.0144	Apolipoprotein A-IV;APOA4;ortholog	APOLIPOPROTEIN A-IV (PTHR18976:SF1)
ENSSSCT00000 048959	-1.2807	0.0470	Transmembrane channel-like protein;TMC5;ortholog	TRANSMEMBRANE CHANNEL-LIKE PROTEIN 5 (PTHR23302:SF5)
ENSSSCT00000 024216	-0.4776	0.0297	Solute carrier family 43 member 2;SLC43A2;ortholog	LARGE NEUTRAL AMINO ACIDS TRANSPORTER SMALL SUBUNIT 4 (PTHR20766:SF2)
ENSSSCT00000 022886	-0.3637	0.0037	Phosphatidylinositol transfer protein alpha;PITPNA;ortholog	PHOSPHATIDYLINOSITOL TRANSFER PROTEIN ALPHA ISOFORM (PTHR10658:SF28)
ENSSSCT00000 019677	0.6836	0.0420	Cytochrome c oxidase subunit 3;MT-CO3;ortholog	CYTOCHROME C OXIDASE SUBUNIT 3 (PTHR11403:SF7)
ENSSSCT00000 006801	0.7319	0.0360	Non-specific serine/threonine protein kinase;SGK3;ortholog	SERINE/THREONINE-PROTEIN KINASE SGK3 (PTHR24356:SF121)
ENSSSCT00000 058817	1.2132	0.0443	Gap junction protein;GJB3;ortholog	GAP JUNCTION BETA-3 PROTEIN (PTHR11984:SF65)

Supplementary Table S5. Genes related to binding proteins

ID	Log2 Fold Change	*p*-value	Gene Name	Panther Family
Zn100 vs LQ100				
ENSSSCT00000003882	-24.1839	0.0000	Phospholipase A(2);PLA2G2A;ortholog	PHOSPHOLIPASE A2, MEMBRANE ASSOCIATED (PTHR11716:SF9)
ENSSSCT00000040161	-2.8331	0.0091	Regulatory factor X7;RFX7;ortholog	DNA-BINDING PROTEIN RFX7 (PTHR12619:SF2)
ENSSSCT00000012564	-2.3531	0.0393	Chromosome 3 open reading frame 67;C3orf67;ortholog	ZGC:162324 (PTHR12458:SF7)
ENSSSCT00000009621	-2.2504	0.0227	Rho-related GTP-binding protein RhoH;RHOH;ortholog	RHO-RELATED GTP-BINDING PROTEIN RHOH (PTHR24072:SF163)
ENSSSCT00000049484	-2.2021	0.0236	Protein S100-G;S100G;ortholog	PROTEIN S100-G (PTHR11639:SF73)
ENSSSCT00000042792	-2.1407	0.0316	Phosphatidylinositol transfer protein membrane associated 2;PITPNM2;ortholog	MEMBRANE-ASSOCIATED PHOSPHATIDYLINOSITOL TRANSFER PROTEIN 2 (PTHR10658:SF41)
ENSSSCT00000055045	-1.9231	0.0475	TBC1 domain family member 7;TBC1D7;ortholog	TBC1 DOMAIN FAMILY MEMBER 7 (PTHR13530:SF3)
ENSSSCT00000066361	-1.2765	0.0184	Ribosomal protein L15;RPL15;ortholog	SUBFAMILY NOT NAMED (PTHR11847:SF20)
ENSSSCT00000000767	-1.0569	0.0026	CD27 antigen precursor;CD27;ortholog	CD27 ANTIGEN (PTHR47496:SF1)
ENSSSCT00000050784	-1.0004	0.0284	Fanconi anemia complementation group D2;FANCD2;ortholog	FANCONI ANEMIA GROUP D2 PROTEIN (PTHR32086:SF0)
ENSSSCT00000057503	-0.8633	0.0357	Cold-inducible RNA-binding protein;CIRBP;ortholog	COLD-INDUCIBLE RNA-BINDING PROTEIN (PTHR15241:SF45)
ENSSSCT00000040645	-0.7488	0.0304	Interferon regulatory factor 8;IRF8;ortholog	INTERFERON REGULATORY FACTOR 8 (PTHR11949:SF7)
ENSSSCT00000063738	-0.6388	0.0231	Transcription factor 3;TCF3;ortholog	TRANSCRIPTION FACTOR E2-ALPHA (PTHR11793:SF7)
ENSSSCT00000043658	-0.6041	0.0113	Tubulin beta chain;TUBB4B;ortholog	TUBULIN BETA-4B CHAIN (PTHR11588:SF247)
ENSSSCT00000043336	-0.5610	0.0480	Copine-1;CPNE1;ortholog	COPINE-1 (PTHR10857:SF2)

ENSSSCT00000054538	-0.4327	0.0183	40S ribosomal protein S9;RPS9;ortholog	40S RIBOSOMAL PROTEIN S9 (PTHR11831:SF5)
ENSSSCT00000036365	-0.4194	0.0073	Ubiquitously expressed prefoldin like chaperone;UXT;ortholog	PROTEIN UXT (PTHR13345:SF4)
ENSSSCT00000028688	-0.4170	0.0372	SERTA domain containing 1;SERTAD1;ortholog	SERTA DOMAIN-CONTAINING PROTEIN 1 (PTHR16277:SF12)
ENSSSCT00000046389	-0.3964	0.0011	GrpE protein homolog;GRPEL1;ortholog	GRPE PROTEIN HOMOLOG 1, MITOCHONDRIAL (PTHR21237:SF25)
ENSSSCT00000059106	-0.3780	0.0458	RNA-binding protein 4;RBM4;ortholog	RNA-BINDING PROTEIN 4 (PTHR23147:SF80)
ENSSSCT00000048373	-0.3470	0.0154	Eukaryotic translation initiation factor 5A;EIF5A;ortholog	EUKARYOTIC TRANSLATION INITIATION FACTOR 5A-1 (PTHR11673:SF34)
ENSSSCT00000054388	-0.3469	0.0337	BRCA2 and CDKN1A-interacting protein;BCCIP;ortholog	BRCA2 AND CDKN1A-INTERACTING PROTEIN (PTHR13261:SF0)
ENSSSCT00000045474	-0.3469	0.0013	Ribosomal protein;RPL10A;ortholog	60S RIBOSOMAL PROTEIN L10A (PTHR23105:SF107)
ENSSSCT00000064349	-0.3453	0.0308	RNA polymerase II subunit D;POLR2D;ortholog	DNA-DIRECTED RNA POLYMERASE II SUBUNIT RPB4 (PTHR21297:SF0)
ENSSSCT00000025029	-0.3355	0.0063	Ribosome biogenesis protein NOP53;NOP53;ortholog	RIBOSOME BIOGENESIS PROTEIN NOP53 (PTHR14211:SF7)
ENSSSCT00000016207	-0.3036	0.0018	40S ribosomal protein S3;RPS3;ortholog	40S RIBOSOMAL PROTEIN S3 (PTHR11760:SF32)
ENSSSCT00000003287	-0.3018	0.0081	Eukaryotic translation initiation factor 3 subunit K;EIF3K;ortholog	EUKARYOTIC TRANSLATION INITIATION FACTOR 3 SUBUNIT K (PTHR13022:SF0)
ENSSSCT00000049763	-0.2901	0.0064	60S acidic ribosomal protein P0;RPLP0;ortholog	60S ACIDIC RIBOSOMAL PROTEIN P0-RELATED (PTHR45699:SF1)
ENSSSCT00000045427	-0.2858	0.0444	Uncharacterized protein;ANAPC11;ortholog	ANAPHASE-PROMOTING COMPLEX SUBUNIT 11 (PTHR11210:SF1)
ENSSSCT00000014925	-0.2657	0.0304	Eukaryotic translation initiation factor 3 subunit G;EIF3G;ortholog	EUKARYOTIC TRANSLATION INITIATION FACTOR 3 SUBUNIT G (PTHR10352:SF0)
ENSSSCT00000040918	-0.2656	0.0295	60S ribosomal protein L5;RPL5;ortholog	60S RIBOSOMAL PROTEIN L5 (PTHR23410:SF12)

ENSSSCT00 000014615	-0.2427	0.0229	40S ribosomal protein S13;RPS13;ortholog	40S RIBOSOMAL PROTEIN S13 (PTHR11885:SF14)
ENSSSCT00 000006622	-0.2106	0.0422	Eukaryotic translation initiation factor 3 subunit E;EIF3E;ortholog	EUKARYOTIC TRANSLATION INITIATION FACTOR 3 SUBUNIT E (PTHR10317:SF0)
ENSSSCT00 000011019	-0.2054	0.0354	Small nuclear ribonucleoprotein Sm D3;SNRPD3;ortholo g	SMALL NUCLEAR RIBONUCLEOPROTEIN SM D3 (PTHR23338:SF17)
ENSSSCT00 000024076	-0.1998	0.0499	60S ribosomal protein L26;RPL26;ortholog	60S RIBOSOMAL PROTEIN L26 (PTHR11143:SF11)
ENSSSCT00 000046697	-0.1606	0.0452	Eukaryotic translation initiation factor 3 subunit H;EIF3H;ortholog	EUKARYOTIC TRANSLATION INITIATION FACTOR 3 SUBUNIT H (PTHR10410:SF3)
ENSSSCT00 000000090	-0.1510	0.0469	Cyclic AMP-dependent transcription factor ATF-4;ATF4;ortholog	CYCLIC AMP-DEPENDENT TRANSCRIPTION FACTOR ATF-4 (PTHR13044:SF2)
ENSSSCT00 000017252	0.2281	0.0430	Glutathione reductase;GSR;orthol og	GLUTATHIONE REDUCTASE, MITOCHONDRIAL (PTHR42737:SF2)
ENSSSCT00 000026750	0.2498	0.0482	Tristetraprolin;ZFP36 ;ortholog	MRNA DECAY ACTIVATOR PROTEIN ZFP36 (PTHR12547:SF122)
ENSSSCT00 000053892	0.3019	0.0265	ADP-ribosylation factor 1;ARF1;ortholog	ADP-RIBOSYLATION FACTOR 1 (PTHR11711:SF350)
ENSSSCT00 000056298	0.3836	0.0191	Class E basic helix-loop-helix protein 40;BHLHE40;ortholo g	CLASS E BASIC HELIX-LOOP-HELIX PROTEIN 40 (PTHR10985:SF3)
ENSSSCT00 000056665	0.3906	0.0487	Parvin alpha;PARVA;orthol og	ALPHA-PARVIN (PTHR12114:SF6)
ENSSSCT00 000018974	0.4091	0.0416	Junction plakoglobin;JUP;orth olog	JUNCTION PLAKOGLOBIN (PTHR45976:SF3)
ENSSSCT00 000053997	0.4404	0.0065	RAS like proto-oncogene B;RALB;ortholog	RAS-RELATED PROTEIN RAL-B (PTHR24070:SF199)
ENSSSCT00 000056807	0.4429	0.0447	MAX dimerization protein 1;MXD1;ortholog	MAX DIMERIZATION PROTEIN 1 (PTHR11969:SF18)
ENSSSCT00 000004193	0.4698	0.0268	Cystathionine gamma-lyase;CTH;ortholog	CYSTATHIONINE GAMMA-LYASE (PTHR11808:SF15)
ENSSSCT00 000044681	0.5222	0.0171	Interleukin 13 receptor, alpha 1;IL13RA1;ortholog	INTERLEUKIN-13 RECEPTOR SUBUNIT ALPHA-1 (PTHR23036:SF89)

ENSSSCT00000053024	0.5948	0.0215	Uncharacterized protein;unassigned;ortholog	GENE MODEL 266, (NCBI) (PTHR24070:SF154)
ENSSSCT00000001722	0.6067	0.0334	Transcriptional enhancer factor TEF-5;TEAD3;ortholog	TRANSCRIPTIONAL ENHANCER FACTOR TEF-5 (PTHR11834:SF0)
ENSSSCT00000044801	0.6997	0.0379	Uncharacterized protein;unassigned;ortholog	IMMUNOGLOBULIN HEAVY VARIABLE 3-11-RELATED (PTHR23266:SF204)
ENSSSCT00000035626	0.7442	0.0269	Aminopeptidase;ANPEP;ortholog	AMINOPEPTIDASE N (PTHR11533:SF172)
ENSSSCT00000066211	0.7692	0.0473	Uncharacterized protein;unassigned;ortholog	PROTEIN HOOK HOMOLOG 3 (PTHR18947:SF38)
ENSSSCT00000063551	0.8325	0.0282	Uncharacterized protein;unassigned;ortholog	IMMUNOGLOBULIN HEAVY VARIABLE 3-11-RELATED (PTHR23266:SF204)
ENSSSCT00000065219	0.8997	0.0426	Tubulin polymerization promoting protein p25 alpha;TPPP;ortholog	TUBULIN POLYMERIZATION-PROMOTING PROTEIN (PTHR12932:SF18)
ENSSSCT00000002650	0.9443	0.0007	Proto-oncogene c-Fos;FOS;ortholog	PROTO-ONCOGENE C-FOS (PTHR23351:SF4)
ENSSSCT00000049254	1.0068	0.0467	Nuclear distribution protein nudE-like 1;NDEL1;ortholog	NUCLEAR DISTRIBUTION PROTEIN NUDE-LIKE 1 (PTHR10921:SF0)
ENSSSCT00000014625	1.2771	0.0112	Cytochrome P450 family 2 subfamily R member 1;CYP2R1;ortholog	VITAMIN D 25-HYDROXYLASE (PTHR24300:SF48)
ENSSSCT00000053543	1.3379	0.0056	Chemokine C-C motif receptor-like 2;CCRL2;ortholog	C-C CHEMOKINE RECEPTOR-LIKE 2 (PTHR10489:SF655)
ENSSSCT00000056394	1.3741	0.0472	Tetratricopeptide repeat domain 23;TTC23;ortholog	TETRATRICOPEPTIDE REPEAT PROTEIN 23 (PTHR14485:SF3)
ENSSSCT00000055882	1.5338	0.0487	Slingshot protein phosphatase 1;SSH1;ortholog	PROTEIN PHOSPHATASE SLINGSHOT HOMOLOG 1 (PTHR45864:SF5)
ENSSSCT00000018449	1.9897	0.0313	Ring finger protein 180;RNF180;ortholog	E3 UBIQUITIN-PROTEIN LIGASE RNF180 (PTHR46717:SF1)
ENSSSCT00000031974	3.5701	0.0398	Metallothionein-1A;MT1A;ortholog	METALLOTHIONEIN-1A (PTHR23299:SF22)
ENSSSCT00000030537	3.6497	0.0274	Histone H4;unassigned;ortholog	HISTONE H4 (PTHR10484:SF163)
**Zn50 vs LQ50**				
ENSSSCT00000015136	-2.7016	0.0041	Epidermal growth factor receptor pathway substrate 15	EPIDERMAL GROWTH FACTOR RECEPTOR SUBSTRATE 15-LIKE 1 (PTHR11216:SF69)

			like 1;EPS15L1;ortholog	
ENSSSCT00 000061466	-2.5828	0.0113	Transcription elongation factor A N-terminal and central domain containing;TCEANC; ortholog	TRANSCRIPTION ELONGATION FACTOR A N-TERMINAL AND CENTRAL DOMAIN-CONTAINING PROTEIN (PTHR11477:SF7)
ENSSSCT00 000035464	-2.5588	0.0028	Uncharacterized protein;LOC1005233 10;ortholog	GUANYLATE-BINDING PROTEIN 6 (PTHR10751:SF62)
ENSSSCT00 000057064	-2.2298	0.0277	Polyhomeotic homolog 3;PHC3;ortholog	POLYHOMEOTIC-LIKE PROTEIN 3 (PTHR12247:SF88)
ENSSSCT00 000037785	-2.1417	0.0210	Sortilin related VPS10 domain containing receptor 2;SORCS2;ortholog	VPS10 DOMAIN-CONTAINING RECEPTOR SORCS2 (PTHR12106:SF9)
ENSSSCT00 000039871	-2.0942	0.0332	Uncharacterized protein;unassigned;or tholog	IMMUNOGLOBULIN HEAVY VARIABLE 3-11-RELATED (PTHR23266:SF204)
ENSSSCT00 000059075	-1.8025	0.0209	Uncharacterized protein;unassigned;or tholog	IMMUNOGLOBULIN HEAVY VARIABLE 3-11-RELATED (PTHR23266:SF204)
ENSSSCT00 000061852	-1.6591	0.0144	Apolipoprotein A-IV;APOA4;ortholog	APOLIPOPROTEIN A-IV (PTHR18976:SF1)
ENSSSCT00 000008980	-1.5834	0.0283	VIP36-like protein precursor;LMAN2L; ortholog	VIP36-LIKE PROTEIN (PTHR12223:SF20)
ENSSSCT00 000029033	-1.5750	0.0224	Synaptotagmin 12;SYT12;ortholog	SYNAPTOTAGMIN-12 (PTHR10024:SF252)
ENSSSCT00 000052291	-1.4563	0.0478	Centromere protein O;CENPO;ortholog	CENTROMERE PROTEIN O (PTHR14582:SF1)
ENSSSCT00 000043753	-1.2366	0.0071	SPT2 chromatin protein domain containing 1;SPTY2D1;ortholog	PROTEIN SPT2 HOMOLOG (PTHR22691:SF8)
ENSSSCT00 000062032	-1.2090	0.0395	Semaphorin 4B;SEMA4B;ortholo g	SEMAPHORIN-4B (PTHR11036:SF14)
ENSSSCT00 000064480	-1.1998	0.0459	Transcription elongation regulator 1;TCERG1;ortholog	TRANSCRIPTION ELONGATION REGULATOR 1 (PTHR15377:SF7)
ENSSSCT00 000037353	-1.1398	0.0007	Uncharacterized protein;unassigned;or tholog	IMMUNOGLOBULIN KAPPA CONSTANT (PTHR23266:SF79)
ENSSSCT00 000002141	-1.1070	0.0055	Promyelocytic leukemia;PML;orthol og	PROTEIN PML (PTHR25462:SF241)
ENSSSCT00 000056748	-1.0832	0.0016	Galectin;LGALS9;ort holog	GALECTIN-9 (PTHR11346:SF80)
ENSSSCT00 000065175	-1.0825	0.0135	Uncharacterized protein;unassigned;or tholog	GOLGI PHOSPHOPROTEIN 3 (PTHR12704:SF3)

ENSSSCT00000002555	-1.0094	0.0108	Arginase;ARG2;ortholog	ARGINASE-2, MITOCHONDRIAL (PTHR43782:SF4)
ENSSSCT00000031638	-0.9830	0.0114	Uncharacterized protein;KDM4A;ortholog	LYSINE-SPECIFIC DEMETHYLASE 4A (PTHR10694:SF32)
ENSSSCT00000001595	-0.8511	0.0173	Novel protein similar to butyrophilin family proteins;LOC100512174;ortholog	BUTYROPHILIN, SUBFAMILY 3, MEMBER A3-RELATED (PTHR24100:SF64)
ENSSSCT00000041931	-0.8153	0.0236	Uncharacterized protein;unassigned;ortholog	IMMUNOGLOBULIN HEAVY VARIABLE 3-11-RELATED (PTHR23266:SF204)
ENSSSCT00000035232	-0.7662	0.0223	Interferon regulatory factor 7;Irf7;ortholog	INTERFERON REGULATORY FACTOR 7 (PTHR11949:SF2)
ENSSSCT00000014648	-0.7431	0.0395	ADM;ADM;ortholog	ADM (PTHR23414:SF3)
ENSSSCT00000025376	-0.6591	0.0037	Basic leucine zipper ATF-like transcription factor 2;BATF2;ortholog	BASIC LEUCINE ZIPPER TRANSCRIPTIONAL FACTOR ATF-LIKE 2 (PTHR23351:SF11)
ENSSSCT00000011756	-0.6423	0.0404	DEAH-box helicase 32 (putative);DHX32;ortholog	PRE-MRNA-SPLICING FACTOR ATP-DEPENDENT RNA HELICASE DHX32-RELATED (PTHR18934:SF88)
ENSSSCT00000016571	-0.6049	0.0209	Hepatic and glial cell adhesion molecule;HEPACAM;ortholog	HEPATOCYTE CELL ADHESION MOLECULE (PTHR12080:SF59)
ENSSSCT00000024524	-0.5898	0.0076	Uncharacterized protein;PPP4R1;ortholog	SERINE/THREONINE-PROTEIN PHOSPHATASE 4 REGULATORY SUBUNIT 1 (PTHR10648:SF8)
ENSSSCT00000064525	-0.4788	0.0459	Arp2/3 complex 34 kDa subunit;ARPC2;ortholog	ACTIN-RELATED PROTEIN 2/3 COMPLEX SUBUNIT 2 (PTHR12058:SF0)
ENSSSCT00000053600	-0.4219	0.0426	Rho GTPase activating protein 17;ARHGAP17;ortholog	RHO GTPASE-ACTIVATING PROTEIN 17 (PTHR14130:SF3)
ENSSSCT00000055437	-0.4102	0.0175	Serine/threonine-protein phosphatase 2A activator;PTPA;ortholog	SERINE/THREONINE-PROTEIN PHOSPHATASE 2A ACTIVATOR (PTHR10012:SF0)
ENSSSCT00000022886	-0.3637	0.0037	Phosphatidylinositol transfer protein alpha;PITPNA;ortholog	PHOSPHATIDYLINOSITOL TRANSFER PROTEIN ALPHA ISOFORM (PTHR10658:SF28)
ENSSSCT00000008551	-0.3458	0.0209	Elongation factor Tu;TUFM;ortholog	ELONGATION FACTOR TU, MITOCHONDRIAL (PTHR43721:SF5)
ENSSSCT00000044242	-0.3178	0.0164	Adhesion regulating molecule 1;ADRM1;ortholog	PROTEASOMAL UBIQUITIN RECEPTOR ADRM1 (PTHR12225:SF0)

ENSSSCT00 000042867	-0.3149	0.0418	Rho GDP dissociation inhibitor alpha;ARHGDIA;ortholog	RHO GDP-DISSOCIATION INHIBITOR 1 (PTHR10980:SF9)
ENSSSCT00 000030514	-0.2753	0.0453	Serine/threonine-protein phosphatase 2A 65 kDa regulatory subunit A alpha isoform;PPP2R1A;ortholog	SERINE/THREONINE-PROTEIN PHOSPHATASE 2A 65 KDA REGULATORY SUBUNIT A ALPHA ISOFORM (PTHR10648:SF2)
ENSSSCT00 000039540	-0.2155	0.0339	Ras-related C3 botulinum toxin substrate 1;RAC1;ortholog	RAS-RELATED C3 BOTULINUM TOXIN SUBSTRATE 1 (PTHR24072:SF105)
ENSSSCT00 000025147	0.4742	0.0323	Histone H2B;HIST1H2BD;ortholog	HISTONE H2B TYPE 1-N (PTHR23428:SF170)
ENSSSCT00 000019682	0.7948	0.0283	NADH-ubiquinone oxidoreductase chain 4;MT-ND4;ortholog	NADH-UBIQUINONE OXIDOREDUCTASE CHAIN 4 (PTHR43507:SF1)
ENSSSCT00 000005070	0.9044	0.0346	Ring finger protein 111;RNF111;ortholog	E3 UBIQUITIN-PROTEIN LIGASE ARKADIA (PTHR16200:SF4)
ENSSSCT00 000001791	0.9390	0.0497	Nuclear transcription factor Y subunit alpha;NFYA;ortholog	NUCLEAR TRANSCRIPTION FACTOR Y SUBUNIT ALPHA (PTHR12632:SF6)
ENSSSCT00 000046065	1.1418	0.0308	High mobility group protein B1;HMGB1;ortholog	SUBFAMILY NOT NAMED (PTHR13711:SF328)
ENSSSCT00 000003507	1.2545	0.0333	Protein lin-7 homolog;LIN7B;ortholog	PROTEIN LIN-7 HOMOLOG B (PTHR14063:SF7)
ENSSSCT00 000032954	2.0028	0.0331	Interleukin-33;IL33;ortholog	INTERLEUKIN-33 (PTHR21114:SF0)
ENSSSCT00 000040161	2.4144	0.0388	Regulatory factor X7;RFX7;ortholog	DNA-BINDING PROTEIN RFX7 (PTHR12619:SF2)
ENSSSCT00 000013823	2.4436	0.0158	Stromal antigen 2;STAG2;ortholog	COHESIN SUBUNIT SA-2 (PTHR11199:SF3)

Supplementary Table S6. Genes related to catalytic activity

ID	Log2 Fold Change	*p*-value	Gene Name	Panther Family
		**Zn100 vs LQ100**		
ENSSSCT0 0000003882	-24.18392	0.00000	Phospholipase A(2);PLA2G2A;ortholog	PHOSPHOLIPASE A2, MEMBRANE ASSOCIATED (PTHR11716:SF9)

ENSSSCT0 0000012564	- 2.35310	0.039 30	Chromosome 3 open reading frame 67;C3orf67;ortholog	ZGC:162324 (PTHR12458:SF7)
ENSSSCT0 0000009621	- 2.25037	0.022 66	Rho-related GTP-binding protein RhoH;RHOH;ortholog	RHO-RELATED GTP-BINDING PROTEIN RHOH (PTHR24072:SF163)
ENSSSCT0 0000055045	- 1.92313	0.047 46	TBC1 domain family member 7;TBC1D7;ortholog	TBC1 DOMAIN FAMILY MEMBER 7 (PTHR13530:SF3)
ENSSSCT0 0000050638	- 1.86996	0.046 23	HtrA serine peptidase 4;HTRA4;ortholog	SERINE PROTEASE HTRA4 (PTHR22939:SF105)
ENSSSCT0 0000000767	- 1.05695	0.002 59	CD27 antigen precursor;CD27;ortholog	CD27 ANTIGEN (PTHR47496:SF1)
ENSSSCT0 0000060206	- 0.94083	0.008 52	SURF1-like protein;SURF1;ortholog	SURFEIT LOCUS PROTEIN 1 (PTHR23427:SF2)
ENSSSCT0 0000014304	- 0.64729	0.008 81	Cytochrome b561 family member A3;CYB561A3;ortholog	CYTOCHROME B ASCORBATE-DEPENDENT PROTEIN 3 (PTHR10106:SF38)
ENSSSCT0 0000030066	- 0.64633	0.022 87	Adenosine deaminase;ADA;ortholog	ADENOSINE DEAMINASE (PTHR11409:SF43)
ENSSSCT0 0000052940	- 0.61962	0.010 89	CCR4-NOT transcription complex subunit 8;CNOT8;ortholog	CCR4-NOT TRANSCRIPTION COMPLEX SUBUNIT 8 (PTHR10797:SF1)
ENSSSCT0 0000008412	- 0.51569	0.031 90	Ribonuclease P protein subunit p20;POP7;ortholog	RIBONUCLEASE P PROTEIN SUBUNIT P20 (PTHR15314:SF1)
ENSSSCT0 0000046389	- 0.39636	0.001 07	GrpE protein homolog;GRPEL1;ortholog	GRPE PROTEIN HOMOLOG 1, MITOCHONDRIAL (PTHR21237:SF25)
ENSSSCT0 0000052185	- 0.37943	0.023 34	Cathepsin D;CTSD;ortholog	CATHEPSIN D (PTHR13683:SF230)
ENSSSCT0 0000054388	- 0.34691	0.033 75	BRCA2 and CDKN1A-interacting protein;BCCIP;ortholog	BRCA2 AND CDKN1A-INTERACTING PROTEIN (PTHR13261:SF0)
ENSSSCT0 0000064349	- 0.34533	0.030 77	RNA polymerase II subunit D;POLR2D;ortholog	DNA-DIRECTED RNA POLYMERASE II SUBUNIT RPB4 (PTHR21297:SF0)
ENSSSCT0 0000044425	- 0.31915	0.049 87	Dihydrouridine synthase 1 like;DUS1L;ortholog	TRNA-DIHYDROURIDINE(16/17) SYNTHASE [NAD(P)(+)]-LIKE (PTHR11082:SF5)
ENSSSCT0 0000016207	- 0.30356	0.001 79	40S ribosomal protein S3;RPS3;ortholog	40S RIBOSOMAL PROTEIN S3 (PTHR11760:SF32)
ENSSSCT0 0000045427	- 0.28581	0.044 41	Uncharacterized protein;ANAPC11;ortholog	ANAPHASE-PROMOTING COMPLEX SUBUNIT 11 (PTHR11210:SF1)
ENSSSCT0 0000015001	- 0.21402	0.049 42	Telomerase RNA component interacting RNase;TRIR;ortholog	TELOMERASE RNA COMPONENT INTERACTING RNASE (PTHR34753:SF1)
ENSSSCT0 0000006146	- 0.18623	0.043 89	Proteasome subunit beta type-7;PSMB7;ortholog	PROTEASOME SUBUNIT BETA TYPE-7 (PTHR11599:SF42)
ENSSSCT0 0000046697	- 0.16058	0.045 22	Eukaryotic translation initiation factor 3 subunit H;EIF3H;ortholog	EUKARYOTIC TRANSLATION INITIATION FACTOR 3 SUBUNIT H (PTHR10410:SF3)
ENSSSCT0 0000017252	0.22807	0.043 03	Glutathione reductase;GSR;ortholog	GLUTATHIONE REDUCTASE, MITOCHONDRIAL (PTHR42737:SF2)

ENSSSCT00000018376	0.27348	0.01958	3-hydroxy-3-methylglutaryl coenzyme A synthase;HMGCS1;ortholog	HYDROXYMETHYLGLUTARYL-COA SYNTHASE, CYTOPLASMIC (PTHR43323:SF4)
ENSSSCT00000011350	0.39216	0.00582	Glutamate dehydrogenase 1, mitochondrial;GLUD1;ortholog	GLUTAMATE DEHYDROGENASE 1, MITOCHONDRIAL (PTHR11606:SF13)
ENSSSCT00000053997	0.44042	0.00651	RAS like proto-oncogene B;RALB;ortholog	RAS-RELATED PROTEIN RAL-B (PTHR24070:SF199)
ENSSSCT00000056464	0.44821	0.03658	Dual specificity protein phosphatase;DUSP5;ortholog	DUAL SPECIFICITY PROTEIN PHOSPHATASE 5 (PTHR10159:SF40)
ENSSSCT00000004193	0.46982	0.02681	Cystathionine gamma-lyase;CTH;ortholog	CYSTATHIONINE GAMMA-LYASE (PTHR11808:SF15)
ENSSSCT00000009288	0.47094	0.01447	Aldose 1-epimerase;GALM;ortholog	ALDOSE 1-EPIMERASE (PTHR10091:SF0)
ENSSSCT00000027607	0.50663	0.01133	Aldehyde dehydrogenase 1 family member A1;ALDH1A1;ortholog	RETINAL DEHYDROGENASE 1 (PTHR11699:SF140)
ENSSSCT00000053024	0.59484	0.02153	Uncharacterized protein;unassigned;ortholog	GENE MODEL 266, (NCBI) (PTHR24070:SF154)
ENSSSCT00000037199	0.60264	0.00962	Dual specificity protein phosphatase;DUSP6;ortholog	DUAL SPECIFICITY PROTEIN PHOSPHATASE 6 (PTHR10159:SF45)
ENSSSCT00000003075	0.62857	0.02655	Corticosteroid 11-beta-dehydrogenase isozyme 2;HSD11B2;ortholog	CORTICOSTEROID 11-BETA-DEHYDROGENASE ISOZYME 2 (PTHR43313:SF2)
ENSSSCT00000007341	0.67544	0.04255	Flavin-containing monooxygenase;FMO5;ortholog	DIMETHYLANILINE MONOOXYGENASE [N-OXIDE-FORMING] 5 (PTHR23023:SF78)
ENSSSCT00000013382	0.67885	0.01927	Ornithine carbamoyltransferase, mitochondrial;OTC;ortholog	ORNITHINE CARBAMOYLTRANSFERASE, MITOCHONDRIAL (PTHR45753:SF3)
ENSSSCT00000045147	0.71343	0.02927	Protein phosphatase, Mg2+/Mn2+ dependent 1H;PPM1H;ortholog	PROTEIN PHOSPHATASE 1H (PTHR13832:SF287)
ENSSSCT00000035626	0.74420	0.02689	Aminopeptidase;ANPEP;ortholog	AMINOPEPTIDASE N (PTHR11533:SF172)
ENSSSCT00000058969	1.02098	0.03695	GMP reductase;GMPR;ortholog	GMP REDUCTASE 1 (PTHR43170:SF3)
ENSSSCT00000055231	1.03997	0.04582	Protein-tyrosine sulfotransferase;unassigned;ortholog	PROTEIN-TYROSINE SULFOTRANSFERASE 2 (PTHR12788:SF6)
ENSSSCT00000014625	1.27713	0.01124	Cytochrome P450 family 2 subfamily R member 1;CYP2R1;ortholog	VITAMIN D 25-HYDROXYLASE (PTHR24300:SF48)
ENSSSCT00000050440	1.85768	0.04775	Microsomal glutathione S-transferase 3;MGST3;ortholog	MICROSOMAL GLUTATHIONE S-TRANSFERASE 3 (PTHR10250:SF17)
ENSSSCT00000018449	1.98967	0.03129	Ring finger protein 180;RNF180;ortholog	E3 UBIQUITIN-PROTEIN LIGASE RNF180 (PTHR46717:SF1)

Zn50 vs LQ50				
ENSSSCT00000016255	-2.70906	0.00403	RAB30, member RAS oncogene family;RAB30;ortholog	RAS-RELATED PROTEIN RAB-30 (PTHR24073:SF408)
ENSSSCT00000035464	-2.55879	0.00284	Uncharacterized protein;LOC100523310;ortholog	GUANYLATE-BINDING PROTEIN 6 (PTHR10751:SF62)
ENSSSCT00000061852	-1.65906	0.01443	Apolipoprotein A-IV;APOA4;ortholog	APOLIPOPROTEIN A-IV (PTHR18976:SF1)
ENSSSCT00000011816	-1.40772	0.02951	Hexosyltransferase;B3GALT2;ortholog	BETA-1,3-GALACTOSYLTRANSFERASE 2 (PTHR11214:SF19)
ENSSSCT00000058969	-1.19064	0.04771	GMP reductase;GMPR;ortholog	GMP REDUCTASE 1 (PTHR43170:SF3)
ENSSSCT00000045468	-1.14623	0.00684	Histo-blood group ABO system transferase;GBGT1;ortholog	GLOBOSIDE ALPHA-1,3-N-ACETYLGALACTOSAMINYLTRANSFERASE 1 (PTHR10462:SF29)
ENSSSCT00000002555	-1.00938	0.01081	Arginase;ARG2;ortholog	ARGINASE-2, MITOCHONDRIAL (PTHR43782:SF4)
ENSSSCT00000031638	-0.98305	0.01141	Uncharacterized protein;KDM4A;ortholog	LYSINE-SPECIFIC DEMETHYLASE 4A (PTHR10694:SF32)
ENSSSCT00000003489	-0.93477	0.04099	Galactoside 2-alpha-L-fucosyltransferase 2;FUT2;ortholog	GALACTOSIDE 2-ALPHA-L-FUCOSYLTRANSFERASE 2 (PTHR11927:SF2)
ENSSSCT00000006932	-0.89043	0.00597	4-trimethylaminobutyraldehyde dehydrogenase;ALDH9A1;ortholog	4-TRIMETHYLAMINOBUTYRALDEHYDE DEHYDROGENASE (PTHR11699:SF228)
ENSSSCT00000044789	-0.80731	0.01832	Succinyl-CoA:3-ketoacid coenzyme A transferase 1, mitochondrial;OXCT1;ortholog	SUCCINYL-COA:3-KETOACID COENZYME A TRANSFERASE 1, MITOCHONDRIAL (PTHR13707:SF30)
ENSSSCT00000061681	-0.79278	0.00707	Argininosuccinate synthase 1;ASS1;ortholog	ARGININOSUCCINATE SYNTHASE (PTHR11587:SF2)
ENSSSCT00000011756	-0.64232	0.04039	DEAH-box helicase 32 (putative);DHX32;ortholog	PRE-MRNA-SPLICING FACTOR ATP-DEPENDENT RNA HELICASE DHX32-RELATED (PTHR18934:SF88)
ENSSSCT00000016571	-0.60487	0.02091	Hepatic and glial cell adhesion molecule;HEPACAM;ortholog	HEPATOCYTE CELL ADHESION MOLECULE (PTHR12080:SF59)
ENSSSCT00000024524	-0.58979	0.00760	Uncharacterized protein;PPP4R1;ortholog	SERINE/THREONINE-PROTEIN PHOSPHATASE 4 REGULATORY SUBUNIT 1 (PTHR10648:SF8)
ENSSSCT00000037199	-0.58239	0.02900	Dual specificity protein phosphatase;DUSP6;ortholog	DUAL SPECIFICITY PROTEIN PHOSPHATASE 6 (PTHR10159:SF45)
ENSSSCT00000065553	-0.57552	0.01641	Uridine phosphorylase;UPP1;ortholog	URIDINE PHOSPHORYLASE 1 (PTHR43691:SF10)

ENSSSCT00000029497	-0.56234	0.01635	7-dehydrocholesterol reductase;DHCR7;ortholog	7-DEHYDROCHOLESTEROL REDUCTASE (PTHR21257:SF38)
ENSSSCT00000057292	-0.56159	0.02472	Sulfotransferase;CHST4;ortholog	CARBOHYDRATE SULFOTRANSFERASE 4 (PTHR10704:SF40)
ENSSSCT00000044432	-0.52325	0.03527	Serine/threonine kinase 24;STK24;ortholog	SERINE/THREONINE-PROTEIN KINASE 24 (PTHR24361:SF406)
ENSSSCT00000003977	-0.50887	0.04196	Sphingomyelin phosphodiesterase acid like 3B;SMPDL3B;ortholog	ACID SPHINGOMYELINASE-LIKE PHOSPHODIESTERASE 3B (PTHR10340:SF25)
ENSSSCT00000053991	-0.47170	0.00615	RAB7A, member RAS oncogene family;RAB7A;ortholog	RAS-RELATED PROTEIN RAB-7A (PTHR24073:SF556)
ENSSSCT00000053600	-0.42187	0.04258	Rho GTPase activating protein 17;ARHGAP17;ortholog	RHO GTPASE-ACTIVATING PROTEIN 17 (PTHR14130:SF3)
ENSSSCT00000055437	-0.41019	0.01755	Serine/threonine-protein phosphatase 2A activator;PTPA;ortholog	SERINE/THREONINE-PROTEIN PHOSPHATASE 2A ACTIVATOR (PTHR10012:SF0)
ENSSSCT00000063535	-0.40673	0.02898	Citrate synthase;CS;ortholog	CITRATE SYNTHASE, MITOCHONDRIAL (PTHR11739:SF8)
ENSSSCT00000011231	-0.37573	0.02691	Pyrophosphatase (inorganic) 1;PPA1;ortholog	INORGANIC PYROPHOSPHATASE (PTHR10286:SF47)
ENSSSCT00000014210	-0.35799	0.04150	Calpain-1 catalytic subunit;CAPN1;ortholog	CALPAIN-1 CATALYTIC SUBUNIT (PTHR10183:SF284)
ENSSSCT00000044242	-0.31781	0.01644	Adhesion regulating molecule 1;ADRM1;ortholog	PROTEASOMAL UBIQUITIN RECEPTOR ADRM1 (PTHR12225:SF0)
ENSSSCT00000042867	-0.31488	0.04177	Rho GDP dissociation inhibitor alpha;ARHGDIA;ortholog	RHO GDP-DISSOCIATION INHIBITOR 1 (PTHR10980:SF9)
ENSSSCT00000003774	-0.27872	0.01623	6-phosphogluconate dehydrogenase, decarboxylating;PGD;ortholog	6-PHOSPHOGLUCONATE DEHYDROGENASE, DECARBOXYLATING (PTHR11811:SF25)
ENSSSCT00000030514	-0.27528	0.04531	Serine/threonine-protein phosphatase 2A 65 kDa regulatory subunit A alpha isoform;PPP2R1A;ortholog	SERINE/THREONINE-PROTEIN PHOSPHATASE 2A 65 KDA REGULATORY SUBUNIT A ALPHA ISOFORM (PTHR10648:SF2)
ENSSSCT00000039540	-0.21554	0.03390	Ras-related C3 botulinum toxin substrate 1;RAC1;ortholog	RAS-RELATED C3 BOTULINUM TOXIN SUBSTRATE 1 (PTHR24072:SF105)
ENSSSCT00000019677	0.68365	0.04200	Cytochrome c oxidase subunit 3;MT-CO3;ortholog	CYTOCHROME C OXIDASE SUBUNIT 3 (PTHR11403:SF7)
ENSSSCT00000019673	0.68503	0.03749	Cytochrome c oxidase subunit 2;COX2;ortholog	CYTOCHROME C OXIDASE SUBUNIT 2 (PTHR22888:SF9)
ENSSSCT00000019679	0.69623	0.03616	NADH-ubiquinone oxidoreductase chain 3;MT-ND3;ortholog	NADH-UBIQUINONE OXIDOREDUCTASE CHAIN 3 (PTHR11058:SF9)
ENSSSCT00000019675	0.72406	0.04397	ATP synthase protein 8;MT-ATP8;ortholog	ATP SYNTHASE PROTEIN 8 (PTHR13722:SF0)

ENSSSCT00000019670	0.73158	0.04251	Cytochrome c oxidase subunit 1;MT-CO1;ortholog	CYTOCHROME C OXIDASE SUBUNIT 1 (PTHR10422:SF18)
ENSSSCT00000006801	0.73190	0.03600	Non-specific serine/threonine protein kinase;SGK3;ortholog	SERINE/THREONINE-PROTEIN KINASE SGK3 (PTHR24356:SF121)
ENSSSCT00000051906	0.73500	0.01345	Ubiquitin carboxyl-terminal hydrolase 34;USP34;ortholog	UBIQUITIN CARBOXYL-TERMINAL HYDROLASE 34 (PTHR24006:SF671)
ENSSSCT00000019682	0.79478	0.02831	NADH-ubiquinone oxidoreductase chain 4;MT-ND4;ortholog	NADH-UBIQUINONE OXIDOREDUCTASE CHAIN 4 (PTHR43507:SF1)
ENSSSCT00000019686	0.83361	0.03718	NADH-ubiquinone oxidoreductase chain 5;MT-ND5;ortholog	NADH-UBIQUINONE OXIDOREDUCTASE CHAIN 5 (PTHR42829:SF2)
ENSSSCT00000019681	0.85735	0.03173	NADH-ubiquinone oxidoreductase chain 4L;MT-ND4L;ortholog	NADH-UBIQUINONE OXIDOREDUCTASE CHAIN 4L (PTHR11434:SF0)
ENSSSCT00000035892	0.88067	0.00438	Putative deoxyribonuclease TATDN1;TATDN1;ortholog	DEOXYRIBONUCLEASE TATDN1-RELATED (PTHR10060:SF15)
ENSSSCT00000005070	0.90442	0.03459	Ring finger protein 111;RNF111;ortholog	E3 UBIQUITIN-PROTEIN LIGASE ARKADIA (PTHR16200:SF4)
ENSSSCT00000025069	0.94736	0.03615	Limb and CNS expressed 1;LIX1;ortholog	PROTEIN LIMB EXPRESSION 1 HOMOLOG (PTHR31139:SF5)
ENSSSCT00000054049	1.88216	0.04542	ST3 beta-galactoside alpha-2,3-sialyltransferase 6;ST3GAL6;ortholog	TYPE 2 LACTOSAMINE ALPHA-2,3-SIALYLTRANSFERASE (PTHR13713:SF8)

Supplementary Table S7. Genes related to molecular function regulators

ID	Log2 Fold Change	*p*-value	Gene Name	Panther Family
**Zn100 vs LQ100**				
ENSSSCT00000055045	-1.92313	0.04746	TBC1 domain family member 7;TBC1D7;ortholog	TBC1 DOMAIN FAMILY MEMBER 7 (PTHR13530:SF3)
ENSSSCT00000000767	-1.05695	0.00259	CD27 antigen precursor;CD27;ortholog	CD27 ANTIGEN (PTHR47496:SF1)
ENSSSCT00000046389	-0.39636	0.00107	GrpE protein homolog;GRPEL1;ortholog	GRPE PROTEIN HOMOLOG 1, MITOCHONDRIAL (PTHR21237:SF25)
ENSSSCT00000054388	-0.34691	0.03375	BRCA2 and CDKN1A-interacting protein;BCCIP;ortholog	BRCA2 AND CDKN1A-INTERACTING PROTEIN (PTHR13261:SF0)
**Zn50 vs LQ50**				
ENSSSCT00000015136	-2.70160	0.00415	Epidermal growth factor receptor pathway substrate 15 like 1;EPS15L1;ortholog	EPIDERMAL GROWTH FACTOR RECEPTOR SUBSTRATE 15-LIKE 1 (PTHR11216:SF69)
ENSSSCT00000061852	-1.65906	0.01443	Apolipoprotein A-IV;APOA4;ortholog	APOLIPOPROTEIN A-IV (PTHR18976:SF1)
ENSSSCT00000024524	-0.58979	0.00760	Uncharacterized protein;PPP4R1;ortholog	SERINE/THREONINE-PROTEIN PHOSPHATASE 4 REGULATORY SUBUNIT 1 (PTHR10648:SF8)
ENSSSCT00000053600	-0.42187	0.04258	Rho GTPase activating protein 17;ARHGAP17;ortholog	RHO GTPASE-ACTIVATING PROTEIN 17 (PTHR14130:SF3)
ENSSSCT00000055437	-0.41019	0.01755	Serine/threonine-protein phosphatase 2A activator;PTPA;ortholog	SERINE/THREONINE-PROTEIN PHOSPHATASE 2A ACTIVATOR (PTHR10012:SF0)
ENSSSCT00000044242	-0.31781	0.01644	Adhesion regulating molecule 1;ADRM1;ortholog	PROTEASOMAL UBIQUITIN RECEPTOR ADRM1 (PTHR12225:SF0)
ENSSSCT00000042867	-0.31488	0.04177	Rho GDP dissociation inhibitor alpha;ARHGDIA;ortholog	RHO GDP-DISSOCIATION INHIBITOR 1 (PTHR10980:SF9)
ENSSSCT00000030514	-0.27528	0.04531	Serine/threonine-protein phosphatase 2A 65 kDa regulatory subunit A alpha isoform;PPP2R1A;ortholog	SERINE/THREONINE-PROTEIN PHOSPHATASE 2A 65 KDA REGULATORY SUBUNIT A ALPHA ISOFORM (PTHR10648:SF2)
ENSSSCT00000006801	0.73190	0.03600	Non-specific serine/threonine protein kinase;SGK3;ortholog	SERINE/THREONINE-PROTEIN KINASE SGK3 (PTHR24356:SF121)

Supplementary Table S8. Genes related to molecular transducer activity

ID	Log2 Fold Change	*p*-value	Gene Name	Panther Family
**Zn100 vs LQ100**				
ENSSSCT00000054388	-0.3469	0.0337	BRCA2 and CDKN1A-interacting protein;BCCIP;ortholog	BRCA2 AND CDKN1A-INTERACTING PROTEIN (PTHR13261:SF0)
ENSSSCT00000044681	0.5222	0.0171	Interleukin 13 receptor, alpha 1;IL13RA1;ortholog	INTERLEUKIN-13 RECEPTOR SUBUNIT ALPHA-1 (PTHR23036:SF89)
ENSSSCT00000053543	1.3379	0.0056	Chemokine C-C motif receptor-like 2;CCRL2;ortholog	C-C CHEMOKINE RECEPTOR-LIKE 2 (PTHR10489:SF655)
**Zn50 vs LQ50**				
ENSSSCT00000062032	-1.2090	0.0395	Semaphorin 4B;SEMA4B;ortholog	SEMAPHORIN-4B (PTHR11036:SF14)
ENSSSCT00000046065	1.1418	0.0308	High mobility group protein B1;HMGB1;ortholog	SUBFAMILY NOT NAMED (PTHR13711:SF328)
ENSSSCT00000032954	2.0028	0.0331	Interleukin-33;IL33;ortholog	INTERLEUKIN-33 (PTHR21114:SF0)

Supplementary Table S9. Genes related to structural molecular activity

ID	Log2 Fold Change	*p*-value	Gene Name	Panther Family
**Zn100 vs LQ100**				
ENSSSCT00000066361	-1.2765	0.0184	Ribosomal protein L15;RPL15;ortholog	SUBFAMILY NOT NAMED (PTHR11847:SF20)
ENSSSCT00000043658	-0.6041	0.0113	Tubulin beta chain;TUBB4B;ortholog	TUBULIN BETA-4B CHAIN (PTHR11588:SF247)
ENSSSCT00000008748	-0.4617	0.0101	ICT1;MRPL28;ortholog	39S RIBOSOMAL PROTEIN L28, MITOCHONDRIAL (PTHR13528:SF2)
ENSSSCT00000054538	-0.4327	0.0183	40S ribosomal protein S9;RPS9;ortholog	40S RIBOSOMAL PROTEIN S9 (PTHR11831:SF5)
ENSSSCT00000064200	-0.3477	0.0156	Ribosomal protein L19;RPL19;ortholog	60S RIBOSOMAL PROTEIN L19 (PTHR10722:SF13)
ENSSSCT00000014851	-0.3333	0.0063	40S ribosomal protein S28;RPS28;ortholog	40S RIBOSOMAL PROTEIN S28 (PTHR10769:SF3)
ENSSSCT00000031015	-0.3273	0.0273	Ribosomal protein L8;RPL8;ortholog	60S RIBOSOMAL PROTEIN L8 (PTHR13691:SF16)
ENSSSCT00000063010	-0.3111	0.0034	60S ribosomal protein L3;RPL3;ortholog	60S RIBOSOMAL PROTEIN L3 (PTHR11363:SF4)
ENSSSCT00000003515	-0.3081	0.0455	40S ribosomal protein S11;RPS11;ortholog	40S RIBOSOMAL PROTEIN S11 (PTHR10744:SF9)
ENSSSCT00000016207	-0.3036	0.0018	40S ribosomal protein S3;RPS3;ortholog	40S RIBOSOMAL PROTEIN S3 (PTHR11760:SF32)
ENSSSCT00000049763	-0.2901	0.0064	60S acidic ribosomal protein P0;RPLP0;ortholog	60S ACIDIC RIBOSOMAL PROTEIN P0-RELATED (PTHR45699:SF1)
ENSSSCT00000041942	-0.2739	0.0372	40S ribosomal protein S19;RPS19;ortholog	40S RIBOSOMAL PROTEIN S19 (PTHR11710:SF0)
ENSSSCT00000015437	-0.2723	0.0457	40S ribosomal protein S23;RPS23;ortholog	40S RIBOSOMAL PROTEIN S23 (PTHR11652:SF45)
ENSSSCT00000005457	-0.2525	0.0151	60S ribosomal protein L4;RPL4;ortholog	60S RIBOSOMAL PROTEIN L4 (PTHR19431:SF0)
ENSSSCT00000027316	-0.2456	0.0258	60S ribosomal protein L29;RPL29;ortholog	60S RIBOSOMAL PROTEIN L29 (PTHR12884:SF18)
ENSSSCT00000014615	-0.2427	0.0229	40S ribosomal protein S13;RPS13;ortholog	40S RIBOSOMAL PROTEIN S13 (PTHR11885:SF14)
ENSSSCT00000015919	-0.2222	0.0182	60S ribosomal protein L27a;RPL27A;ortholog	60S RIBOSOMAL PROTEIN L27A (PTHR11721:SF3)
ENSSSCT00000043834	-0.2132	0.0097	40S ribosomal protein S20;RPS20;ortholog	RIBOSOMAL PROTEIN S20 (PTHR11700:SF8)
ENSSSCT00000024076	-0.1998	0.0499	60S ribosomal protein L26;RPL26;ortholog	60S RIBOSOMAL PROTEIN L26 (PTHR11143:SF11)
**Zn50 vs LQ50**				
ENSSSCT00000064525	-0.4788	0.0459	Arp2/3 complex 34 kDa subunit;ARPC2;ortholog	ACTIN-RELATED PROTEIN 2/3 COMPLEX SUBUNIT 2 (PTHR12058:SF0)

ENSSSCT00000065139	-0.3094	0.0330	Actin, cytoplasmic 1;ACTB;ortholog	ACTIN, CYTOPLASMIC 1 (PTHR11937:SF192)

Supplementary Table S10. Genes related to transcription regulator activity

ID	Log2 Fold Change	*p*-value	Gene Name	Panther Family
**Zn100 vs LQ100**				
ENSSSCT00000040161	-2.8331	0.0091	Regulatory factor X7;RFX7;ortholog	DNA-BINDING PROTEIN RFX7 (PTHR12619:SF2)
ENSSSCT00000012564	-2.3531	0.0393	Chromosome 3 open reading frame 67;C3orf67;ortholog	ZGC:162324 (PTHR12458:SF7)
ENSSSCT00000040645	-0.7488	0.0304	Interferon regulatory factor 8;IRF8;ortholog	INTERFERON REGULATORY FACTOR 8 (PTHR11949:SF7)
ENSSSCT00000063738	-0.6388	0.0231	Transcription factor 3;TCF3;ortholog	TRANSCRIPTION FACTOR E2-ALPHA (PTHR11793:SF7)
ENSSSCT00000043336	-0.5610	0.0480	Copine-1;CPNE1;ortholog	COPINE-1 (PTHR10857:SF2)
ENSSSCT00000028688	-0.4170	0.0372	SERTA domain containing 1;SERTAD1;ortholog	SERTA DOMAIN-CONTAINING PROTEIN 1 (PTHR16277:SF12)
ENSSSCT00000000090	-0.1510	0.0469	Cyclic AMP-dependent transcription factor ATF-4;ATF4;ortholog	CYCLIC AMP-DEPENDENT TRANSCRIPTION FACTOR ATF-4 (PTHR13044:SF2)
ENSSSCT00000056298	0.3836	0.0191	Class E basic helix-loop-helix protein 40;BHLHE40;ortholog	CLASS E BASIC HELIX-LOOP-HELIX PROTEIN 40 (PTHR10985:SF3)
ENSSSCT00000018974	0.4091	0.0416	Junction plakoglobin;JUP;ortholog	JUNCTION PLAKOGLOBIN (PTHR45976:SF3)
ENSSSCT00000056807	0.4429	0.0447	MAX dimerization protein 1;MXD1;ortholog	MAX DIMERIZATION PROTEIN 1 (PTHR11969:SF18)
ENSSSCT00000001722	0.6067	0.0334	Transcriptional enhancer factor TEF-5;TEAD3;ortholog	TRANSCRIPTIONAL ENHANCER FACTOR TEF-5 (PTHR11834:SF0)
**Zn50 vs LQ50**				
ENSSSCT00000061466	-2.5828	0.0113	Transcription elongation factor A N-terminal and central domain containing;TCEANC;ortholog	TRANSCRIPTION ELONGATION FACTOR A N-TERMINAL AND CENTRAL DOMAIN-CONTAINING PROTEIN (PTHR11477:SF7)
ENSSSCT00000057064	-2.2298	0.0277	Polyhomeotic homolog 3;PHC3;ortholog	POLYHOMEOTIC-LIKE PROTEIN 3 (PTHR12247:SF88)
ENSSSCT00000002141	-1.1070	0.0055	Promyelocytic leukemia;PML;ortholog	PROTEIN PML (PTHR25462:SF241)

ENSSSCT000 00031638	-0.9830	0.011 4	Uncharacterized protein;KDM4A;ortholo g	LYSINE-SPECIFIC DEMETHYLASE 4A (PTHR10694:SF32)
ENSSSCT000 00035232	-0.7662	0.022 3	Interferon regulatory factor 7;Irf7;ortholog	INTERFERON REGULATORY FACTOR 7 (PTHR11949:SF2)
ENSSSCT000 00001791	0.9390	0.049 7	Nuclear transcription factor Y subunit alpha;NFYA;ortholog	NUCLEAR TRANSCRIPTION FACTOR Y SUBUNIT ALPHA (PTHR12632:SF6)
ENSSSCT000 00046065	1.1418	0.030 8	High mobility group protein B1;HMGB1;ortholog	SUBFAMILY NOT NAMED (PTHR13711:SF328)
ENSSSCT000 00040161	2.4144	0.038 8	Regulatory factor X7;RFX7;ortholog	DNA-BINDING PROTEIN RFX7 (PTHR12619:SF2)
